# Abstracts of papers presented at the ISLAR (International Symposium on Laboratory Automation and Robotics) 1998

**DOI:** 10.1155/S1463924699000115

**Published:** 1999


Journal of Automated Methods & Management in Chemistry, Vol. 21, No. 3 (May–June 1999) pp. 65–105

### Abstracts of papers presented at the ISLAR (International Symposium on Laboratory Automation and Robotics) 1998

The 16th International Symposium on Laboratory Automation and Robotics provided the largest number of technical presentations ever offered at this meeting. State-of-the-art development in laboratory automation and robotics were reflected in the symposium programme which included papers and posters on all aspects of the technology—drug discovery research, data handling and data management, chemical analysis, bio-analytical applications, managing laboratory automation, dissolution testing, pharmaceutical analysis, automation and combinatorial chemistry, validating automated methods and advanced topics. Several new workshops and discussion sessions were included and activated, and provided even more interactive communication.

Although the programme was very comprehensive, the Symposium was designed to provide time for both formal and informal exchange of information. The technical presentations were organized into concurrent sessions with grouped papers on related topics.

Abstracts for each paper (except two that were not available when going to press) and each poster are included here and the proceedings of the Symposium will be published as a CD-Rom early in 1999. Full presentations of several of these papers will appear in later editions of this journal.

### Integrating drug discovery technologies

*James A. Bristol, Parke-Davis Pharmaceutical Research, Ann Arbor, MI, USA*

The pharmaceutical industry today is entering a new era wherein a number of new and enabling technologies are maturing simultaneously. These enabling technologies include combinatorial chemistry, ultra-high-throughput screening, genomics, pharamacogenomics, proteomics, data management, high-throughput methodology for toxicology, and ADME parameters. In addition, new and improved automation techniques will have a dramatic impact on optimization of these new technologies. At the same time, the unprecedented productivity and financial success of new pharmaceutical agents and the effect of mergers leading to vastly larger single companies have created unprecedented demands for continued improvements in productivity that will be translated directly to increased sales and profits. A further factor is the increasing trend to outsource key discovery and development activities to biopharmaceutical companies and contract research organizations.

These enabling technologies have the potential to transform pharmaceutical discovery into a highly efficient technological advanced process which will demand appropriate resourcing and integration for optimal efficiency. These developments are the challenges for drug discovery scientists in the future who, in addition to having established expertise within their own scientific disciplines, will necessarily have to work effectively at the interface of these intersecting technologies and scientific disciplines.

### Coordinated development of multi-site/multi-functional analytical robotics applications: challenges and visions

*E. F. McNiff, Bristol-Myers Squibb, Princeton, NJ, USA*

The analytical development laboratory provides multiple opportunities for increasing productivity through automation and robotics. Today's generation of analytical scientists has arrived on the job accustomed to using and developing automated approaches to their work. They expect automation, and where it does not exist, will seek to add it. There are many approaches to automation of an analytical technique. The approach can vary depending on the scope and impact of the application, but also whether it is being developed by an analytical scientist or an engineer. A challenge exists in maintaining the automation creativity of individual scientists, while providing a mechanism for an integrated and coordinated approach to development within a multi-site, multi-functional organization. From a diverse mix of analytical chemists, biochemists, process control scientists, software developers and mechanical engineers, have come a wide range of automated systems. An overview of these systems will be provided with emphasis on the successes and challenges of the various development approaches which were utilized. The ongoing evolution into the current team approach to automation development will be presented.

### Quality control: organization, cycle times, compliance and efficiency

*J. M. Hawkins, Janssen Pharmaceutica, Titusville, NJ, USA*

Understanding the contributions that the laboratory can make in product/process development, process improvement, market surveillance and general business is key to the pharmaceutical business today. Poor laboratory practice yields compliance issues, increased cost, increased cycle time and delayed product introductions. In my presentation, I hope to prompt discussion and gain agreement and recognition of the laboratory's contribution in meeting the expectations of our pharmaceutical customers. I will cover key areas of customer satisfaction, the role of the quality control and quality assurance laboratories, measures of accountability and progress, and an example of how our robotics program is helping Janssen meet our goals.

### Automation in the lab: does the tail wag the dog?

*Simon Jones, Genetics Institute, Cambridge, MA, USA*

Intensive automation of repetitive processes in the lab is viewed as an attractive panacea for several reasons. First is the assumption that removal of the human element in process leads to consistently accurate data sets in a shorter period of time. Second, and deriving from the first, is that scientists are 'freed' to focus their talents on more creative pursuits. Third, head count can be stabilized and even reduced. Based on this rationale, a virtuous circle is established that pleases all parties in a biopharmaceutical endeavour.

However, in reality several issues were overlooked and unanticipated problems have arisen. First, and the most serious, is the displacement of the bottleneck in sample handling to processes up- and downstream of the automated procedure, e.g. data analysis, compound inventory and secondary assays in high-throughput screening. Second, the daily function of automated units requires repetitive and accurate operation. This frequently leads to the 'burn out' phenomenon and turnover of personnel. Third, a substantial financial investment is required not only in equipment and bulk materials, but in training personnel. Fourth, a whole new breed of scientist has developed to adapt existing bench processes or create new ones for automated systems.

These issues and their resolution in small molecule drug discovery will be presented.

### The future of high-throughput screening in drug discovery

*John Babiak, Robotics and Automation, Wyeth-Ayerst Research, Princeton, NJ and Pearl River, NY, USA*

High-throughput screening (HTS), in conjunction with genomics, combinatorial chemistry and molecular modelling, provides pharmaceutical companies with therapeutic opportunities never before possible. HTS involves using automated equipment to test a large number of samples against a novel molecular or cellular target to identify a reasonable number of active molecules in a timely fashion. Under the best of circumstances, the active molecules identified by HTS can be the chemical leads which will be optimized into important new marketed drugs. Because most drug discovery efforts fail to result in a marketed drug, the primary pressure on HTS is to do more—more targets and more test samples—to increase the likelihood of success.

Many HTS groups within pharmaceutical companies have done more through the use of automation, improved process design and hard work. It is now common for HTS groups to test several hundred thousand samples against 20 or more novel targets each year. These levels of productivity are roughly 10-fold greater than what was typically achieved just 2 or 3 years ago. The popular trends towards assay miniaturization, industrial-strength automation and new detection technologies further extend the view that more is better. However, finding more leads for more novel targets does not necessarily shorten the time to develop a chemical lead into a drug or increase the likelihood of success for an individual project.

In the future, therefore, an emphasis needs to be placed on using HTS resources and expertise to improve the quality of chemical leads, not simply find more of them. Concepts in automation, data management and streamlined assay design which have been developed within the HTS environment will be applied to other aspects of drug discovery, e.g. target validation and secondary screening. Broader adoption of the tools of HTS may be able to achieve improvements in drug discovery time lines and cost savings which cannot occur through more and faster HTS.

### Libraries of molecules, screening and optimization of hits: a cost-effective approach

*Nigel R. A. Beeley, Amylin Pharmaceuticals, San Diego, CA, USA*

As a product-oriented biotechnology company we need inexpensive solutions for acquiring libraries of molecules, introducing screening robotics, handling data and automating chemistry when compared to the larger pharmaceutical companies and technology-oriented start-up companies. We now have access to over 250 000 compounds for screening purposes from the following: (i) pharmacophore-based selective purchasing from several purveyors of compounds; (ii) risk-sharing arrangements giving access to relatively large numbers of compounds; and (iii) participation in a combinatorial chemistry consortium. We have put in place the necessary informatics and screening robotics to identify hits and analyse data from the above. In addition, we have re-tooled our chemistry effort to focus on optimization of hits, making extensive use of parallel synthesis. Some recent examples from our anti-obesity and anti-diabetic programs will be presented.

### The HTS infrastructure—the right tools for the task

*Mark Beggs, Janssen Research Foundation, Belgium*

High-throughput screening (HTS) is a key component of the pharmaceutical lead identification process at Janssen Research Foundation. Over recent years, the pharmaceutical industry has experienced significant increases in the throughput capabilities of its HTS functions. In those companies where HTS has been effectively deployed, it is now possible to screen the entire corporate compound collection against a pharmacological target within a timescale of several weeks to a few months. This capability has been realized, not as a result of the purchase of any one particular piece of hardware, but rather through the development of a truly effective HTS infrastructure which matches the needs of the parent organization. Central to this development is the need to understand how to effectively combine the use of the different types of hardware available to the HTS specialist.

The use of both modular workstations and single-arm robotic systems has underpinned most HTS groups' operations. Recent advances in the field of multiple-arm robotic systems and dedicated automation systems offer even further potential for increasing productivity. This talk will describe our experience with the use of such technologies within Janssen Research Foundation's HTS group.

### Implementation of analytical technologies in a pharmaceutical development organization—looking into the next millennium

*Nigel North, Pharmaceutical Technologies, SmithKline Beecham Pharmaceuticals, Harlow, Essex, UK*

Managing the implementation of new technology in a pharmaceutical development environment has provided challenges and opportunities to obtain the benefits from technologies, e.g. laboratory automation. Successful application of new techniques requires a dedicated resource. Within our department, this resource was initially a single person that has since evolved into a team dedicated to the investigation and development of robotics and non-invasive analytical techniques, including near-infrared. Pharmaceutical development is an important interface between research and commercial manufacturing. In research, the success of genomics and combinatorial chemistry will result in a significant increase in the number of development compounds. This increase combined with the desire of commercial manufacturing to move towards parametric release puts an emphasis on the need for rapid analytical methods. Some ideas on the techniques that will be required to meet these goals will be described together with their impact on automation.

### Laboratory automation in pharmaceutical development: into the new millennium

*Stephen Scypinski, Analytical Development, The R. W. Johnson Pharmaceutical Research Institute, Raritan, NJ, USA*

Incredibly, the use of laboratory automation and robotics in pharmaceutical analysis and drug development is almost 20 years old. Those of us who have attended ISLAR over the past years have heard success stories, trials and tribulations of automating many types of pharmaceutical dosage from assays. More recently, the automation wave has hit the drug discovery area quite hard, and it is amazing to see the rapid implementation of automation and robotics in areas, e.g. high-throughput screening and combinatorial chemistry. If one compares the 'ramps of utilization' between discovery and development in its utilization of automation, the question to be asked is... WHY?

I have asked myself the aforementioned question and have tried to answer it by factoring in the rigidities we in development are confronted with, e.g. regulatory methods, cGMP compliance, method system and computers contributing to the non-usage of robotics in drug development and pharmaceutical analysis, their contribution is almost certainly small. One must begin to ask the question: If we in development are indeed gong to integrate robotics into our repertoire of analytical tools and techniques, what needs to be done to make this happen?

Recently joining a new organization helps one establish perspective and gives a new beginning and new ideas. One approach being tried is to firmly 'build automation in' when designing methods and sample preparation schemes. This approach accomplishes several things:

(1) shifting the paradigm of conventional sample preparation to that compatible with automation (i.e. homogenization);
(2) translating/transferring manual methods to an automated system becomes more facile;
(3) persons utilizing both see more equivalency between them, and hence usage is more readily accepted.

Using several examples, aspects of automation applied to the challenges we all face as we move into the 21st century will be highlighted.

### Automated analytical equipment for routine testing used in the quality control labs

*H. Emmerich, S. Kurschat, W. Seifert, A. Siegle and M. Fischer, Byk Gulden, Lomberg Chemische Fabrik GmbH, Singen, Germany*

In the past 10 years, laboratory automation (previously used primarily in clinical–chemical analysis) became a more important tool for routine testing in pharmaceutical quality control. The use of automated or semi-automated laboratory equipment can decrease the lab flow-through time of the samples for release testing of raw materials and finished products as well as for stability studies, thus saving manual capacity for other analytical work. Therefore, to improve efficiency, the first automated equipment was introduced into the quality control labs of Byk Gulden in the early 1990s. These instruments were prototype robotic systems which automated the release testing of an X-ray contrast medium and stability studies of enteric coated tablets (including assay and purity testing). After the implementation and qualification phase (approx. 1 year for each system), the equipment has been used over a period of about 4 years for routine testing and benchmarking studies. The results show that the lab flow through time for the samples of one contrast medium batch can be cut in half using the automated method compared to the corresponding manual procedure (8 h instead of 16 h). The number of batches that can be tested per day, week or month is no longer limited by the working hours of the analysts, but only by the capacity of the equipment. Due to these positive experiences, a further automated instrument was ordered in January 1998 for the analysis of a new X-ray contrast medium produced for the USA market. The first economical data for this robotic system show the same significant drop in the lab flow-through time of the samples as m.a. for the other robotic systems (10 h instead of 20 h).

### Developing and managing the automated laboratory

*Muhammad Albrakeh, Iltifat Hasan and Richard Ashley, Barr Laboratories, Pomona, NY, USA*

The development of new laboratory automated methods is at the forefront of R&D and QC. This technology enables higher output, reduces waste and promotes safer laboratory practices, while cutting down operating costs. Managing such a lab can be both challenging and rewarding. Barr Labs will discuss first hand the process of managing the automated laboratory. Topics include IQ/OQ, preventive maintenance and calibration, necessary training for scientists and systems users, management suggestions, first-hand experiences, benefits and pitfalls, and tips which will carry us into the next millenium. Equipment discussed include: Zymark MultiDose Plus, Zymark Tablet Processing Workstations (TPW II), Zymark BenchMate II, and Zymark—fully automated—Batch Dissolution Systems with USP apparatus I & II for complete on-line dissolution testing, utilizing both UV and HPLC methods.

### Succeeding with bioanalytical automation in a matrix environment

*Glenn A. Smith and R. A. Biddlecombe, Glaxo Wellcome, Research Triangle Park, NC, USA*

The International Division of Bioanalysis and Drug Metabolism (BioMet) at Glaxo Wellcome is currently comprised of distinct departments, each serving a specific role in the drug development process. These departments are organized into smaller project teams whose members collectively contain the necessary skill sets required to advance compounds through their portion of the development process and onto the next department. Expertise in areas, e.g. bioanalysis, HPLFC, LCMS and automation are no longer centralized but instead are spread throughout the divisional groups, creating a flat and balanced organizational structure.

The strengths, weaknesses, opportunities and threats of such a matrix structure will be cited, and the implications they have on laboratory automation will be discussed. Specific examples of successful communication, teamwork and personnel development strategies, as well as automation technologies and systems that enhance efficiency in a matrix environment, will be shared.

### Fully automated SPE-LC-MS-MS: the continuing story

*R. A. Biddlecombe and S. Pleasance, Department of International Bioanalysis, Glaxo Wellcome, Ware, Hertfordshire, UK*

We have previously reported on the application of a fully automated stand-alone system for SPE in the 96-well format. Based around a Zymark XP robot, these systems use a cooled storage carousel, which acts as both a warehouse for all the labware and a refrigerated storage unit for the sample extracts, and a custom-built SPE station. The SPE station incorporates a vacuum manifold, a RAS\RAM station for rapid reagent addition and a nine- or 12-port solvent switching valve. This station is used to perform all the vacuum steps, conditioning, washing and elution steps. A robotic sample processor is used to carry out sample loading.

Further developments of the system to allow automated sample concentration, reconstitution of sample extracts and protein precipitation in the 96-well format will be presented.

Working with the manufacturer, we have developed an on-line system linking the Zymark 96-well SPE system to a triple quandrapole mass spectrometer. A fully scriptable version of sample control coupled with the ability to share files between the Zymark PC and the mass spectrometer Macintosh allow a dynamic link to be set up.

### Automation of the analysis of Loracarbef in human plasma

*T. A. Mihalski and A. Witkowski, BAS Analytics, 2701 Kent Avenue, West Lafaryette, IN, 47906, USA*

A recent clinical study required the quantitation of the antibiotic Loracarbef in human plasma. An HPLC-UV method for this compound base on manual solid phase extraction had already been published [Kovach, P. M., Lantz, R. J., and Brier, G., *J. Chrom.*, **567** (1991), 129–139], but it was believed that automation of the procedure would increase sample throughput. Therefore, a direct transfer of the manual method to an automated one was conducted utilizing the RapidTrace™ SPE Workstation. The resulting assay was fully validated and found to be very robust. Use of the RapidTrace™ proved to increase both assay precision and sample throughput.

### Comparison of automated liquid–liquid extraction with solid phase extraction using a Zymark Rapid Trace™ instrument

*Thierry D. Mann, Dianne Boland and Michael F. Burke, Department of Chemistry, University of Arizona, Tucson, AZ, USA*

The use of LC/MS in a wide variety of analyses has caused a rethinking of the need for highly selective sample preparation techniques in some laboratories. It is generally accepted that solid phase extraction (SPE) provides greater potential selectivity than liquid–liquid extraction; however, the mass spectrum provides a great deal of selectivity in many cases. If selectivity is not required, the LLC experiment can be argued to be simpler and less expensive. Another of the advantages that solid phase extraction (SPE) has over liquid–liquid extraction (LLE) has been its amenability to automation. Recent advances in laboratory automation equipment though, have made it possible to perform simple liquid–liquid extractions in parallel. The purpose of this presentation is to do a direct comparison of SPE and LLE using automation provided by the RapidTrace system.

Two formats for performing liquid–liquid extractions have been investigated. The first utilizes a thin membrane that is transparent to organic solvents. The control of the flow rate of the solvents employed in the experiment by the RapidTrace allows the issues of solvent densities, membrane wetting and contact time with the sample to be addressed. The second format for liquid–liquid extraction involves the use of a mechanically supported stationary liquid phase. In this case, a porous solid support, e.g. diatomaceous earth, is used to support a thin layer of sample solution. The extracting solvent is then applied to the cartridge and passes through, while extracting the analytes. Alternatively, the extracting solvent is used to saturate the surface of the sorbent and the sample is then loaded onto the column. The analytes of interest are then eluted using an appropriate solvent. It should be noted that this approach, like SPE, has effectively two separate extraction steps. The extraction of acidic, neutral and basic analytes from serum and urine will be presented with an emphasis on analysis time, extraction selectivity and other factors that affect recovery.

### Experience with a workstation-based multiple parallel solid phase synthesis system co-developed by Zymark Corporation and ZENECA Pharmaceuticals

*Richard A. Wildonger and James B. Campbell, Chemical Technology Section, Lead Discovery Department, ZENECA Pharmaceuticals, Wilimington, DE, USA*

In response to the rapid advances in the high-throughput synthesis arena and in consideration of our requirements, we entered into a collaboration with Zymark Corporation aimed at co-developing a workstation-based solid phase multiple parallel synthesis system. Thus, we set out to automate selected processes associated with multiple parallel solid phase synthesis, e.g. diversity reagent preparation, synthesis (reagent addition, washing, mixing and short-term incubation), off-line incubation and product cleavage. From the start, we held to the tenet that it is the chemistry that should serve to define the shape of the automation platform rather than the converse.

We will describe the modules completed to date which include the diversity reagent preparation station, the synthesis platform, incubation station and cleavage station. The synthesis platform is unique in that it exemplifies three modes of liquid addition, i.e. plumbed-serial, plumbed-parallel and robot-mediated plumbed-serial. Furthermore, spent reagents, impurities and soluble by-products are removed by filtration using positive pressure differentials rather than vacuum, thus avoiding atmospheric contamination. Features of the synthesis platform include:

- ninety-six reaction positions (4 × 24 position reaction blocks);
- parallel addition of reaction solvents and common reagents;
- robot-mediated delivery of diversity reagents (100);
- gentle agitation by intermittent sparge with inert gas;
- heating to 150 °C or cooling to −75 °C with an additional reflux zone for vapour condensation at reflux;
- resin bed washing by parallel means;
- quick disconnects for rapid removal of reactor blocks for remote processing;
- all wetted materials are Teflon, PPS, glass or stainless steel;
- small footprint is accommodated in a standard fume hood.

A variety of chemistries have been performed to date. These procedures will be described and compared with manual methods.

### Generation of a directed metalloprotease library by solid phase synthesis

*J. M. Salvino, New Lead Generation Department, Rhone Poulenc Rorer, Collegeville, PA, USA*

Metalloproteases are an important class of enzymes involved in processing inactive precursor proteins to their biological active forms. Selective inhibitors of specific metalloproteases have led to important therapeutic agents. The design and synthesis of a library of novel, low molecular weight hydroxamic acids has been undertaken. This library was constructed on solid phase using a seven-step sequence, wherein the final hydroxamic acid formation was performed using a novel in-house developed linker. Biological testing against numerous metalloproteases has revealed potent, selective inhibitors. Details concerning the scope and limitations of the synthesis, challenges in automation and biological activity of the inhibitors will be discussed.

### Automated synthesis at AgrEvo

*Brian A. Moloney, Automated Synthesis and Combinatorial Chemistry, AgrEvo UK, Chesterford Park, Saffron Walden, Essex, UK*

This paper will describe the different approaches taken to automated synthesis at AgrEvo in both England and Germany. The approach taken in the UK is to have a series of workstations, centred around a Zymark robotic platform. Separate evaporation and chromatography workstations are used to maximize the sample throughput. In Frankfurt, an ISRA robot with on-line evaporation and chromatography is used to minimize operator involvement. Each of these approaches will be discussed in detail in the presentation.

### Parallel organic synthesis and combinatorial drug discovery at Affymax

*V. Antonenko, Affymax Research Institute, Santa Clara, CA, USA*

### A multi-task consumer products robotics automation system

*Jonathan J. Zieman, Paul L. Morabito, Ramasamy Tamilarasan, The Dow Chemical Company, Midland, MI, USA; Paul D. Hazelwood, The Dow Chemical Company, Sarnia, Ontario, Canada, and Kevin Wagers, S. C. Johnson Wax, Racine, WI, USA*

An automation system to prepare and test cleaning formulations according to large and complex experimental designs was implemented in the former Dow Brands Business group. Temperature-dependent phase separation and continuous refractive index measurement as well as microemulsion room temperature turbidity may be performed on the prepared formulations, or for product or competitive analysis. Quaternary amine and bleach titrations can be performed using a Brinkmann Titrator integrated into the system. The system will handle 10 formulation solutions, including relatively viscous solutions, and prepare and/or test up to 240 samples per run. All formulations are individually specified allowing maximum flexibility in experimental design. A Visual Basic™ Graphic User Interface (GUI) oversees the Zymark System V™ controller, and provides data input and output with Excel® Spreadsheets by Object Linking and Embedding (OLE). Custom automation peripherals along with the required Easylab™ code were produced, including a SciLog Expert™ Pump to provide one high viscosity solution delivery channel. Further tests considered were viscosity, conductivity and competitive analysis for per cent solids. The system has significant positive economic impact and allows much more rapid formulation of cleaning products in addition to reducing the tedium of repetitive manual operations.

### Development of a fully automated analysis for the riboflavin vitamers in unfortified foods

*L. F. Russell, L. Brooks and K. McRae, Agriculture & Agri-Food Canada, Kentville, Nova Scotia, Canada*

Demands for more and better nutrient composition data and the necessity to test large numbers of food samples in order to generate representative nutrient values is fuelling the need for laboratory automation. A fully automated method has been developed for the simultaneous analysis of riboflavin and its coenzymes, flavin mononucleotide (FMN) and flavin adenine dinucleotide (FAD) in unfortified foods. It combined robotic extraction with HPLC quantitation. The robotic–HPLC method was compared with the HPLC quantitation preceded with a manual extraction. The manual extraction and HPLC separation have been shown to provide total ribvoflavin results that are comparable with the standard AOAC International fluorometric method. The two methods were compared using foods that are significant dietary sources of riboflavin, e.g. liver, beef steak, eggs and milk.

The results from the fully automated method compared favourably with those obtained using manual extraction. The robotic extraction was faster than its manual counterpart and permitted determinations to be conducted under conditions that protected the riboflavin vitamers from degradation and loss during analysis. As a result, the automated method is suitable for unattended analysis of the large numbers of samples that are needed to provide a representative estimate of the mean vitamer content and its inherent variability in unfortified food products.

### Development and validation of an automated method for the determination of total fat in food products by acidic hydrolytic extraction and quantitative gas chromatography

*Lee Pieta and Steven J. Salata, Nabisco, East Hanover, NJ, USA*

In its Nutritional Labeling and Education Act of 1993 (NLEA), the US Food and Drug Administration (USFDA) defined total fat as the sum of fatty acids expressed as triglycerides. Virtually all packaged foods regulated by the USFDA are required to carry specific nutritional information on total and saturated fat. The total fat method adopted by the Association of Official Analytical Chemist (AOAC) is essentially a combination of two official methods. A total lipid extract is obtained by first digesting the sample with an acid. The released fats are extracted into ethyl and petroleum ethers. After gently evaporating off the ethers, the lipids are saponified and methylated. The total fatty acid content is determined using capillary gas chromatography. Nabisco, in partnership with Zymark Corporation, has implemented a laboratory automation system as the solution to a time-consuming and technique-oriented procedure.

### A method for the determination of amprenavir in human plasma and serum using a Zymark/Tecan robotics system and LC/MS/MS detection in a 96-well format

*Cindy M. Rawls and Glenn A. Smith, International Bioanalysis, GlaxoWellcome, Research Triangle Park, NC, USA*

A sensitive, robust, high-throughput method for the determination of amprenavir, an investigational HIV protease inhibitor, was developed and validated for bioanalytical support and clinical trials. This protein precipitation method utilizes an integrated system consisting of a Zymate II robot, a Tecan RSP 8051, a reagent addition station (RAS) with a 25-port solvent selection valve for all liquid additions, and a custom microplate vortex and centrifuge.

Amprenavir and its internal standard (a stable isotope of amprenavir) are detected on an API-300 triple-quadrupole mass spectrometer by atmospheric pressure chemical ionization tandem mass spectrometry (APCI/MS/MS) in positive ion multiple reaction monitoring (MRM) mode. The assay has a dynamic range of 10–5000 ng $ml^{-1}$.

The robotics system processes two 96-well plates in less than 2 h including sample dilutions. Data presented from two clinical trials in which more than 3500 samples were analysed over 20 analytical runs demonstrated excellent accuracy and precision in both serum and plasma.

### Validation test plan for the Packard Multi PROBE™ robotics system

*Clark V. Williard, Jason D. Morse and Thomas D. Oglesby, Taylor Technology, Princeton, NJ, USA*

A fully automated 96-well solid phase extraction method was developed for use in analytical support of GLP studies. A Packard MultiPROBE™ robotics system was used to automate the preparation and extraction of standards, QCs and study samples. As part of the validation scheme for the method and in accordance with regulated guidelines for computer-controlled systems, a validation plan was devised and executed. A validation protocol was created which included a description of the system, a test plan, reference to related SOPs and documentation of testing. It addressed requirements of user verification of the vendor systems and source codes, change control procedure, application system documentation, and the system's physical analytical performance parameters including position control and volumetric pipetting. The validation plan and results will be presented. Additionally, the plan will be discussed with respect to fulfilment of recent regulatory mandates and guidelines.

### Direct injection of plasma for high-throughput drug metabolism HPLC analysis

*Art Sims, Joe Takarewski and Peter Duriga, Cohesive Technologies, MA, USA*

A major time-limiting step in measuring drugs in plasma is sample preparation. Steps necessary to remove the drugs of interest from plasma prior to injection into a HPLC typically require more time than the analysis. Recent advances in automation of solid phase extraction (SPE) have greatly reduced the amount of hands-on time required for sample clean up, but even automated, this process is still prohibitive to high sample throughput. This poster describes new, dynamic flow technology that allows for direct injection of plasma samples and new levels of sample throughput.

Turbulent flow chromatography, and the resulting convective mass transfer, has removed the time-consuming sample clean up traditionally required in plasma drug analysis by allowing direct injection of plasma onto a specialized column. Relatively large (50 μ), phase-coated particles retain the drugs of interest, while the plasma is eluted to waste. The drugs can then be quickly eluted and measured by UV/VIS or mass-specific detectors. This poster will show data from direct plasma injection and sample analysis cycle times as short as 90 s.

### Method optimization approaches using 96-well SPE disk plates

*David J. Ehresman, Greg Lawless and David A. Wells, 3M Filtration Products, St. Paul, MN, USA*

Ninety-six-well solid phase extraction (SPE) in microtitre plate format offers a rapid sample preparation approach for the analysis of drugs and metabolites in biological fluids. Beyond the gains achieved by the microtitre format and parallel processing of 96 samples, the 3M Empore disk technology (90% particles: 10% PTFE, w/w) provides an additional gain in throughput. The use of disks, instead of traditional packed particle beds, allows for smaller solvent volumes and the ability to eliminate the dry-down and reconstitution step prior to analysis. The conditions under which disks should optimally be used are slightly different than with traditional products. This presentation will discuss proper usage and disk plates for maximal efficiency. Also, optimization of disk-based SPE assays will be discussed with emphasis on developing a robust, high-throughput assay. In particular, these aspects of method optimization are discussed in detail: conditioning solvent volumes, vacuum considerations, sample pretreatment options, sample dilution, wash solvent strategies to eliminate proteins, elution solvent optimization and automation considerations. The 96-well plate format is ideal for examining multiple parameters within a single plate. A method can literally be optimized within a single plate, greatly reducing the time required for method development.

### Automated liquid/liquid extractions using Varian Chem Elut™ cartridges and a Zymark XP robotics system

*John R. Alianti, Christine M. Grosse and Glenn A. Smith, International Bioanalysis, Glaxo Wellcome, Research Triangle Park, NC, USA*

Diatomaceous earth (DE) is a silica-based substance composed of the exoskeletons of fossilized unicellular algae called diatoms. As the diatoms died, their skeletal remains blanketed the floors of ancient sea bottoms, lakebeds and lagoons. Today, DE is excavated, processed and purified for use in a variety of products including insecticides and filtration systems.

One important application for DE is in the pharmaceutical industry. Because DE is extremely hydrophilic, aqueous-based polar drug compounds and their metabolites readily adsorb to this matrix. The compounds can then be extracted from the matrix using an immiscible solvent, e.g. ethyl acetate, methylene chloride or chloroform.

At Glaxo Wellcome, several automated LC/MS/MS and HPLC bioanalytical assays have been validated using this procedure because it offers numerous advantages over conventional solid phase and liquid/liquid extractions. This poster describes the advantages and disadvantages of this technique, the system layout, the extraction process, automated vial filling and future trends, e.g. 96-well plate technology.

### Automated 96-well SPE-LC/MS/MS for high-throughput bioanalysis in a contract lab: problems and solutions

*Luca C. Matassa and Patricia E. Paterson, Maxxam Analytics, Mississauga, Ontario, Canada*

Fast bioanalytical analysis techniques, e.g. LC/MS/MS, require equally fast, or faster, sample preparation techniques to keep pace. Automated 96-well SPE with LC/MS/MS has been used successfully in our laboratory for 4 years and has provided significant advantages over manual techniques. There are, however, some specific problems which must be solved in order to take full advantage of the technique. Both problems and solutions of using automated 96-well SPE will be discussed in this presentation.

Major components include 96-well SPE plate (Waters, 3M, Varian), a robotic liquid handling system (Packard Multiprobe 104 or 208), and an LC/MS/S and autosampler (PE-Sciex API III-plus and Hitachi 7250).

Automated 96-well SPE is best applied in high-volume bioanalytical labs. It eliminates rate-limiting steps of manual column preparation and liquid transfers by using 96-well plates coupled to robotic handling of samples, liquids and extracts, thereby providing enhanced throughput and accuracy. On the downside, the technique is semi-automated, requiring manual intervention at some points. Process re-engineering/staff training and purchase of expensive hardware and consumables is required. The automation must also be adaptable to various tubes sizes/configurations and sample matrices from multiple sources.

### Fast on-line Prospekt® SPE/HPLC/MS/MS method for the quantification of methylphenidate in human plasma (potassium oxalate)

*Robert Masse, Christian Lemoyne and Claude Amestoy, Phoenix International Life Sciences, St-Laurent, Quebec, Canada, Daniel Carazzato, Bristol-Myers Squibb (Canada), St-Laurent, Quebec, Canada, Alain Carrier, Triangle (Canada), Montreal, Quebec, Canada, and Denis Lessard, Bioanalytical Innovation, Levis, Quebec, Canada*

Methylphenidate is a mild CNS stimulant with effects similar to that of amphetamine and is frequently prescribed for children with attention-deficit hyperactivity. A fast automated SPE/HPLC/MS/MS method was developed to quantify methylphenidate in human plasma at 0.100 ng/ml. Human plasma (100 ml) containing methylphenidate was diluted with water, extracted with a fully automated on-line solid phase sample clean-up and injection system (Varian Prospekt® 9200 SPE system). Separation and quantification were performed with HPLC/MS/MS. The resultant total run time was 3 min. A full validation was completed, linearity was observed in the range of 0.100–10 ng/ml ($r > 0.9997$). Observed recoveries for methylphenidate at 0.300 ng/ml, 3.999 ng/ml and 7.998 ng/ml were 72.0%, 66.8% and 68.7%, respectively. The between-batch CV% for QC samples at 0.100 ng/ml, 0.300 ng/l, 3.999 ng/ml and 7.998 ng/ml were 7.6%, 5.0%, 4.0% and 3.5%, respectively. Further validation and stability data will be presented.

### Automated analyses of three selected calcium channel blockers: fetodipine, nifedipine and nisoldipine by the Phoenix PhASSETS® extraction robot coupled with gas chromatography

*Carl Dourambels, René Michaud, Johanne Bouchard, Isabelle Barbeau, Alain Arseneault and Robert Massé, Phoenix Life Sciences, Montréal, Quebec, Canada*

The Sample Preparation and Automation (SPA) and Gas Chromatography (GC) departments at Phoenix International Life Sciences have jointly developed a set of unique methods for the analysis of selected calcium channel blockers (CCBs), i.e. felodipine, nifedipine and nidoldipine, by gas chromatography either coupled to a mass-selective detector (MSD) or an electron capture detector (ECD). The three drugs are structurally similar, having close chemical and physical properties, and tend to react in similar ways when exposed to the same extraction conditions. Our unique approach in using automated extraction was PhASSETS® (Phoenix Automated Simple Sample Extraction Treatment System), an automated extraction system composed of a liquid pipetting station and a special extraction cartridge. This system was designed and developed at Phoenix International Life Sciences. This multi-purpose, automated extraction system has been responsible for the validation of over 60 analytical methods, using different means of detection, e.g. HPLC, HPLC/MS, HPLC/MS/MS, GC, GC/MS and immunochemistry assays. The PhASSETS® extraction system makes use of the liquid/liquid extraction theory (adsorption/desorption) coupled with a specially treated solid support cartridge.

The extraction of these drugs from the biological matrix (human plasma/EDTA) can be accomplished with a speed of 120 samples in 1.5 h, leaving the analysts free to perform other duties. Statistical analysis on between-batch quality control samples led to the following results.

| | Felodipine | | | |
|---|---|---|---|---|
| | QC D 40.44 pg/ml | QC A 121.32 pg/ml | QC B 3.033 pg/ml | QC C 6.066 pg/ml |
| Mean | 43.311 | 124.485 | 3.0743 | 6.3292 |
| SD | 4.3202 | 5.9277 | 48.6741 | 172.5209 |
| % CV | 10.0 | 4.8 | 1.6 | 2.8 |
| Nominal (%) | 107.1 | 102.6 | 101.4 | 101.1 |
| Recovery (mean)(%) | | 96.95 | | |
| *N* | 10 | 10 | 9 | 9 |

| | Nifedipine | | | |
|---|---|---|---|---|
| | QC D 0.51 ng/ml | QC A 1.52 pg/ml | QC B 50.54 ng/ml | QC C 70.75 ng/ml |
| Mean | 0.455 | 1.561 | 52.662 | 70.621 |
| SD | 0.0724 | 0.1137 | 2.1257 | 2.2034 |
| % CV | 15.9 | 7.3 | 4.0 | 3.1 |
| Nominal (%) | 89.3 | 102.7 | 104.2 | 99.8 |
| Recovery (mean)(%) | | 84.13 | | |
| *N* | 6 | 6 | 6 | 6 |

| | Nisoldipine | | | |
|---|---|---|---|---|
| | QC D 0.050 ng/ml | QC A 0.149 ng/ml | QC B 2.980 ng/ml | QC C 5.961 ng/ml |
| Mean | 0.494 | 124.485 | 3.1586 | 6.0734 |
| SD | 0.00312 | 5.9277 | 0.14956 | 0.18579 |
| % CV | 6.3 | 4.8 | 4.7 | 3.1 |
| Nominal (%) | 98.8 | 102.6 | 106.0 | 101.9 |
| Recovery (mean)(%) | | 85.23 | | |
| *N* | 6 | 5 | 6 | 6 |

Felodipine and nisoldipine were analysed by GC/MSD, while nifedipine was analysed by GC/ECD. The maximum run times for these analyses were 6 min per sample. Analytical batches typically numbered 95 samples. Chromatography systems were very stable, and no contamination or interference at analyte retention times was found in all blank plasma samples tested. The use of the PhASSETS® extraction system coupled with GC was found to be ideal for these analyses.

### The joint automated analysis of loratadine and descarboethoxy loratadine (major metabolite) by Phoenix PhASSETS® extraction system and liquid chromatography coupled to tandem mass spectrometry

*René Michaud, Carl Dourambeis and Robert Massé, Phoenix International Life Sciences, Montréal, Quebec, Canada*

The Sample Preparation and Automation (SPA) department at Phoenix International Life Sciences has developed a unique method for the joint determination of loratadine and its major metabolite, descarboethoxy loratadine, from human plasma by LC/MS/MS at a quantitation limit of 50 pg/ml. PhASSETS® (Phoenix Automated Simple Sample Extraction Treatment System), an internally developed extraction system has been used to validate this method and over 60 other analytical methods for HPLC, HPLC/MS, GC, GC/MS and immunochemistry departments at Phoenix. This system makes use of liquid/liquid extraction theory (adsorption/desorption) on a solid support termed 'thin-film liquid/liquid extraction'. The PhASSETS robot uses multiple liquid/liquid additions to help selectively improve the recovery of the analyte(s) from the biological matrix. The following results were obtained.

| | Loratadine | | | |
|---|---|---|---|---|
| | QC A ng/ml | QC B ng/ml | QC C ng/ml | QC D |
| Actual concentration | 0.1506 | 6.0240 | 7.5300 | |
| Mean | 0.14926 | 5.70400 | 7.20208 | |
| SD | 0.010128 | 0.235934 | 0.288902 | |
| (%) CV | 6.8 | 4.1 | 4.0 | 9.4 |
| Nominal (%) | 99.1 | 94.7 | 95.6 | 102.2 |
| *N* | 21 | 22 | 22 | 24 |

| | Descarboethoxy loratadine | | | |
|---|---|---|---|---|
| | QC A ng/ml | QC B ng/ml | QC C ng/ml | QC D |
| Actual concentration | 0.1583 | 6.3303 | 7.9129 | 0.0528 |
| Mean | 0.16966 | 6.04556 | 7.56163 | 0.05645 |
| SD | 0.018167 | 0.572729 | 0.531295 | 0.006091 |
| (%) CV | 10.7 | 9.5 | 7.0 | 10.8 |
| Nominal (%) | 107.2 | 95.5 | 95.6 | 106.9 |
| *N* | 21 | 22 | 22 | 22 |

Recovery of both compounds from (sodium EDTA) human plasma was 91.5% for loratadine and 84.9% for descarboethoxy loratadine.

The use of Sciex API-III LC/MS/MS®, coupled with Waters Alliance® injector, led to 2 min run time per sample, with no contamination or interference at the drug's retention times, determined on 10 different blank plasma samples. The use of a simple mobile phase and analytical HPLC column also tends to minimize all variations on the different systems, thus making this a rugged and robust method for all hands involved in the process.

### Evaluation of 96-well solid phase extraction (SPE) technology using methotrexate and aminopterin as drug probes both off-line and using the TOMTEC Quadra 96 automated workstation

*Carl Dourambeis, Rigas Voyatzis, Trang Nguyen and Robert Massé, Phoenix International Life Sciences, Montreal, Quebec, Canada*

Ninety-six-well technology has been investigated using methotrexate and aminopterin as drug and internal standard (IS) probes, respectively. The flow characteristics and regeneration of the 96-well extraction blocks showed differences from manufacturer to manufacturer. Extraction solvent effects at various levels during drug extraction showed the dependence of the recovery on the step in which different solvents were employed. Variable volumes of sample loaded during the extraction showed very little dependence on the sample volume used. Mechanical and chemical weakness in the extraction manifolds and extraction blocks are discussed. Results will show a comparison between various vendors' products as well as optimization techniques leading to the development of a 96-well method for the isolation and purification of methotrexate and the internal standard, aminopterin. HPLC and LC/MS/MS are used as detection systems. Results of this work are presented.

### High-throughput analysis of drugs from biological matrices with a water-wettable sorbent in a 96-well extraction plate

*Yung-Fong Cheng, Laura Bean, Robert Bonin, Ed Bouvier, Mark Capparella, Pam Iraneta, Uwe D. Neue, and Dorothy J. Phillips, Waters Corporation, Milford, MA, USA*

Sample preparation is required to maximize the quality of the analytical results and to minimize the downtime of expensive, high-overhead analytical tools e.g. LC/MS/MS systems. Historically, liquid–liquid extraction and protein precipitation have been the bottleneck in laboratory productivity as they are not easy to automate. Presently, solid-phase extraction (SPE) has been proven to be more readily automated for high sample throughput. The most common high-throughput sample preparation is solid-phase extraction in a 96-well format. The enabling technology for 96-well plates is the single reversed-phase sorbent that can be used to extract all drug classes from biological fluids. A genetic sorbent and a simple SPE method are the keys to automating the analyses for pharmacokinetics studies in drug discovery and the clinical trial samples in drug development.

This paper shows that using the generic Oasis™ HLB sorbent, in a 96-well format, can increase sample preparation productivity. With a simple and well thought out SPE method development strategy using the polymeric Oasis™ sorbent, we were able to obtain excellent results for a wide range of compounds. Recoveries greater than 85% and variability less than 5% were obtained on Oasis™ HLB extraction plates for basic compounds (e.g. antidepressants and their metabolites) and polar drugs (e.g. procainamide and ranitidine). More importantly, because the Oasis™ HLB extraction sorbent is universal, one general method was used for extracting many different drugs covering a wide polarity range.

### High-throughput extraction with a novel polymeric solid-phase sorbent for screening drug candidates

*Judy L. Carmody, Pamela Iraneta, Yung-Fong Cheng, Dorothy Phillips and Uwe Neue, Waters Corporation, Milford, MA, USA*

Rapid and reproducible quantitation of basic and polar drug compounds from biological matrices has historically been a challenging, time-consuming endeavour. Necessary sample preparation techniques used to concentrate or clean up matrices before HPLC of GC analysis have proven to be either tedious and labour intensive, or irreproducible. Presently, the most commonly used technique is reversed-phase solid phase extraction using porous silica bonded with $C_{18}$. The major disadvantage of employing this sorbent with basic compounds, e.g. doxepin, prevents complete elution of basic compounds and results in low, variable results.

Highly reproducible and uncomplicated SPE methods were developed for these types of basic drug compounds covering a wide polarity range using the novel polymeric Oasis® HLB sorbent. Recoveries greater than 90% and reproducibilities less than 5% RSD were realized even with using the Oasis® HLB sorbent in 96-well plate format for high throughput. Most importantly, because Oasis® HLB sorbent is a water-wettable polymer, these results were achieved without concern as to whether or not the sorbent ran dry. Straightforward method development techniques aimed at offering solutions to problems associated with the sample preparation of drugs in biological matrices will be presented.

### Integrated biological sample preparation in the 96-well format

*Min Chang, Jack Conrad, Enid Quinn, Matthew Rieser, Tong Shen, Willium LaBeau, Bahaa Qasawa and Maury Chu, Abbott Laboratories, Abbott Park, IL, USA*

In order to gain the full benefit of parallel sample processing in the 96-well format, every step in a sample preparation scheme must be compatible and preferable all in the same format. At Abbott Laboratories, integrated sample preparation in the 96-well format was used to prepare plasma and urine samples. With the exception of the initial sample transfer, ordinary steps in sample preparation, e.g. the addition of internal standard and sample treatment reagents, mixing, solid phase and liquid–liquid extraction, evaporation (under nitrogen or by vacuum), reconstitution, sample clarification (by centrifuge or filtration) and HPLC injection were all performed in the 96-well format. Several pieces of small equipment were designed and custom built in-house to achieve the best efficiency. Typical sample preparation times for solid phase extraction were reduced to approximately 2.5 h per 96 samples from approximately 5.5 h. Typical sample preparation times for liquid/liquid extraction were reduced to approximately 2.5 h per 96 samples from approximately 6 h.

### Biological sample preparation by on-line solid phase extraction

*Min Chang, Tong Shen and Maury Chu, Abbott Laboratories, Abbott Park, IL, USA*

Two types of unattended on-line solid phase extraction procedures were developed. The first procedure uses a restricted surface reverse phase cartridge to extract the analytes from the plasma or urine. Proteins and other interfering components were washed off with an aqueous mobile phase. After the initial wash, analytes were eluted into the analytical column by the analytical mobile phase which was also used for the chromatographic separation. The extraction cartridge was regenerated after the analytes eluted into the analytical column. The same extraction cartridge was used for the entire analytical run. The other procedure utilized an OSP-2A (JM Science) sample preparation unit. Each sample was extracted onto a separate extraction cartridge (may be re-used) after activation and conditioning. The extraction procedure was essentially the same as the previously described procedure. However, as the sample extraction and the elution and regeneration of the extraction cartridge may occur in parallel, extensive cartridge washing and drying can be performed without increasing analysis time. A system with four sample preparation solvents and air drying will be presented. Both procedures were used to support clinical and toxicity studies.

### Operational qualification of a BenchMate tablet processing workstation (TWP)

*John Leedes, Whitehall Robins, Richmond, VA, USA*

Recently, Whitehall Robins Healthcare Pharmaceutical Research and Development obtained a TPW. This workstation was the first fully automated sample preparation device brought into our laboratory for routine NDA product analysis. To satisfy GMP requirements, it was necessary to develop a qualification and calibration scheme to ensure that this unit was functioning properly. A protocol was written and approved which describes the operational qualification (OQ) testing of the automated device. The resulting document, described in this poster, satisfies all GMP requirements and serves as the calibration method in our metrology program.

### Integrating high-throughput technology into separation science

*Adam M. Fermier, Norberto A. Guzman and Stephen Scypinski, Analytical Development Department, The R. W. Johnson Pharmaceutical Research Institute Raritan, NJ, USA*

The term 'high-throughput' has become synonymous with the streamlining of processes in the laboratory. Up to now, the application of high-throughput technology has, for the most part, been restricted to drug discovery. Indeed, high-throughput screening (HTS) has revolutionized the manner in which new molecular entities (NMEs) are derived from positive assay screens. In the area of pharmaceutical development, whose mission is to transform an NME candidate into a marketable product, there is a continual quest for increased productivity, in order to minimize the total development time between candidate selection and the filling of the appropriate regulatory documentation to apply for product registration.

With this mindset, the analytical laboratory is expected not only to analyse an increased number of samples with a high degree of accuracy and precision, but is also expected to develop and validate methodology, conduct physiochemical characterization studies, etc. in a time-constrained fashion. Pharmaceutical analysts rely heavily upon separation science to conduct their daily activities; however, separations are by definition one of the slowest of analytical techniques. In our laboratory, we have begun to apply the concepts of high-throughput technology to chemical separations. In this regard, we have constructed an instrument capable of parallel processing up to eight simultaneous samples utilizing capillary array technology.

The reasons for our choice of capillary technology were several. First, a capillary array instrument would be capable of performing both chromatographic and electrophoretic separations, thereby rendering it more versatile. Secondly, capillary separations are faster and do not utilize large volumes of reagents or sample. Finally, they conserve space. The capillary array instrument described here consists of eight columns running in parallel. Detection is accomplished by monitoring the absorbance of the eight channels simultaneously with a fibre optic system. The system is controlled via a personal computer interface. The design, testing and demonstrated usage of the system will be presented in this poster.

### Development and validation of an automated method for dissolution testing of a multi-component antiviral drug using the Zymark MultiDose Workstation

*Toni M. Terry, Glaxo Wellcome, Research Triangle Park NC, USA*

The Zymark MultiDose Dissolution Workstation has been used as a standard platform for automated dissolution testing with USP Apparatus II conditions for solid dosage forms of pharmaceutical products. Early versions of the MultiDose system allowed for both off-line and online testing by chromatographic and spectroscopic measurement methods. Subsequent manipulation of the data had to be performed manually or using spreadsheets. This approach could prove especially tedious for multicomponent products. The latest version (2.1) employs the MultiLink Excel-based software for data quantitation. Additionally, version 2.1 has an interface with the Hewlett-Packard (HP) 8453 UV Spectrometer. The HP software contains programs for manipulating data from single and multiple measurements. This poster will discuss the process by which a multi-component analysis (MCA) method is developed and validated using the MultiDose 2.1-HP8453-HP ChemStation UV software platform. A detailed description of the hardware and software configuration is presented, along with analytical validation results for the product tested.

### A vision-based system for automatic determination of antibiotic content

*Margarida Machado, Departamento de Electrónica, INETI, Azinhaga dos Lameiros, Estrada do Paço do Lumiar, 1699 Lisboa Codex, Portugal*

Antibiotic content determination is essential not only for controlling the quality of pharmaceutical and other products in which the antibiotics are used as additives, but also to ensure compliance with legislation. The microbiological method for evaluation of antibiotic contents is based on the observation of specific microorganisms' reaction to these chemical substances. This reaction takes the form of inhibition halos that appear in the culture medium, around circular cavities where the antibiotic was introduced. As the halos' diameters are directly proportional to the antibiotic concentration, it is possible to evaluate sample contents by comparing their responses with those from standards with known activity.

Traditionally, the reading of the halos' diameters is performed manually by human operators with the help of a digital calliper. These readings involve repetitive manipulations which, besides being time consuming and tedious, demand great precision.

The vision system presented evaluates antibiotic content from images of Petri dishes with inoculated culture and eight inhibition halos, corresponding to four different dilutions of the standard and the sample.

The automatic determination method has four phases. In the first one, an initial segmentation is performed trying to obtain the halo's contours. This segmentation is executed taking into account global information obtained from the histogram. Then a validation analysis is carried out in order to identify the halos and eliminate error situations, like, e.g. the consideration of a halo's hole instead of the halo itself. In this analysis, factors, e.g. the object's circularity, area and centre position are considered. Situations of non-detection are also analysed. After this second phase, a third one is triggered in case of possible errors detected. It consists of a second segmentation, performed in restricted areas and using local intensity information, followed by another error analysis. For the halos identified, a diameter determination procedure is executed. This procedure tries to minimize the fact that halos are not 'perfect' circles, by averaging the distances between opposite contour points situated at equally spaced angular intervals. Finally the content is evaluated using the diameter's values according to the standard norm.

Besides the automatic capability, the system also offers the possibility of diameter's correction, either automatic, semi-automatic or manual, so that the user can observe the effects of content value. An automatic transfer of data to a worksheet is also available.

The tests performed on two antibiotics, Zinc Bacitracin and Tylosin, for three different content values showed a mean difference inferior to 2.5% of the expected theoretical value, between content values obtained from manually determined diameter values and those given by the system in automatic mode without corrections. A standard deviation inferior to 2.5% was observed. The values with automatic correction are of the same order.

### A robotic system for skin permeation test in pharmaceutical research development

*P. Marty, C. Faure and F. Laroche, Institut de Recherche Pierre Fabre, Rue Jean Rostand, 31319 Labege Innopole, Cedex, France*

*In vitro* skin permeation tests using static diffusion cells are commonly used in pharmaceutical development to assess the delivery of dermal or transdermal dosage forms through biological (skin) or artificial membranes. Among the various models described, the Franz cells model is currently still the reference even though it was initially designed for manual sampling (using a syringe), at different time intervals, of the receptor medium. For this type of implementation, a robotic system has been developed to automate sampling for U5 Franz cells arranged on three thermostatically controlled trays. The advantage of this system is that it provides increased capacity, productivity and reproducibility of the basic permeation test, while still using the standard cell model. Improved reproducibility is obtained through better temperature regulation, better control of the displaced volumes (sampled quantities and replaced volumes) and, more generally, by creating uniform manual operations for the methods currently being performed.

### Validation of an automated waste shot workstation—determination of weight of actuation

*Mark J. Richards and Robin V. Platt, Rhône-Poulenc Rorer, Holmes Chapel, Cheshire, UK*

The process of validating methods for use on an automated workstation (MDI FD-10 Station, InnovaSystems, Pennsauken, NJ, USA) for the handling of waste shots from metred dose inhalation products is best managed when broken into various tasks (i.e. shot weight equivalency, medication delivery equivalency). This poster will present the activities related to determination of average weight of actuation (as per cent of target weight); comparing shot weights of manually actuated MDI canisters with canisters actuated on two different MDI FD-10 Stations. Shot weight data are presented as a per cent of target shot weight.

Using two MDI FD-10 Stations, 60 canisters, containing 200 puffs each, were actuated. Weights were recorded prior to placement in the MDI FD-10 and at every 10 actuations hence. Manual shot weights were collected using three analysts actuating 30 MDI canisters containing 200 puffs each. Each analyst actuated 10 canisters each and recorded shot weights prior to actuation and every 10 actuations hence.

Using statistical tests, the data collected show that the MDI FD-10 Stations are statistically equivalent with manual actuation and weighing of MDI canisters. A summary of the data is shown in the table.

| | Means of actuation | | |
|---|---|---|---|
| | Manual | MDI FD-10#1 | MDI FD-10#2 |
| Mean shot weight (% of target) | 97.5 | 97.9 | 98.3 |
| SD | 3.352 | 1.451 | 1.307 |
| CV | 3.4 | 1.5 | 1.3 |
| *n* | 598 | 600 | 599 |
| Min. shot weight (% of target) | 80 | 90.9 | 95.4 |
| Max. shot weight (% of target | 109 | 104 | 103 |

The results of automated actuation of MDI canisters using the MDI FD-10 Station reflect lower coefficient of variances (CV) and standard deviations (SD) when compared with manual actuation. In addition to the statistical equivalence of the results, the tremendous reduction in risk of repetitive strain injuries when using the MDI FD-10 Station when compared to manual actuation of MDI canisters is important. Not only will the MDI FD-10 Station significantly reduce the user's exposure to repetitive strain injury-related activity, but it will also provide the analyst's need to shake and fire multiple MDI canisters needed for testing.

### Automated sample preparation of Roxifiban tablets: transfer of a manual method to a robotic workstation

*William Shamrock and David K. Lloyd, Pharmaceutical R&D, The DuPont Merck Pharmaceutical Co., Experimental Station, Wilmington, DE, USA*

Automation offers obvious advantages for the preparation of tablets prior to analysis by HPLC, including unattended operation, minimization of human intervention and an electronic audit trail. However, significant effort has to be put in up front to develop and validate an automated method, particularly if it is required to closely follow an existing manual method. Here, method transfer for Roxifiban, a fibrinogen receptor antagonist, will be discussed. A Zymark Tablet Processing Workstation II (TPWII) was used for all robotic sample preparations. Manual methods for composite assay and degradation products testing of a tablet formulation were transferred to the TPWII. The method involved dissolving the dosage form by homogenization, filtration of the homogenate, dilution of the filtrate and transfer to autosampler vials. Obstacles to a quick transfer included limitations in the volume capabilities of the TPWII, poorly soluble analytes, and proper conditioning of the transfer lines and filter. After resolving these issues, a validated method was achieved. Spiked recoveries were from 99.4% to 101.1% (RSDs < 0.5%). A cross-validation between automated and manual assay methods was compared by Westlake analysis giving a 0.7% calculated interval of the 95% confidence level. Carry-over was 0.07% after 20 sample preparations at the highest tablet strength.

### Automation of Andersen impaction testing of pressurized metered dose inhalers

*Jonathan Faulkes, Rob Whyard and Astra Chamwood, Loughborough, UK; Malcolm Smith, Novi Systems, Maidstone, UK*

The Anderson impactor is an established device for characterizing the size distribution of particles in an air flow. This manual apparatus has become the standard technique for determining the particle size distribution of the delivered dose from pharmaceutical inhalation products. As a manual method it is time consuming, resource intensive and repetitive. Automating Andersen testing has been a high priority within the pharmaceutical industry.

A collaborative project between Astra Charnwood and the automation company Novi Systems has led to the successful automation of Andersen testing. This was a very difficult automation project due to the design of the manual device. To aid acceptance by regulatory authorities, the internal construction of the Andersen impactor could not be significantly altered.

The philosophy of the automated system is discussed along with the operation and parameters that may be altered to develop an automated test method. Validation data are also presented showing the equivalence between auto and manual determinations. The potential resource savings and reduction on the variability of results from going from a manual to an automated system are also discussed.

### Dissolution bath sampling and analysis optimization

*Charles R. Frey, Gilson, Middleton, WI, USA*

Dissolution testing guidelines require precise and accurate control of temperature, stirring configuration and sampling time. Due to the extended length of tests and sampling time requirements, sampling and analysis procedures are typically automated. Depending on the level of automation that can be achieved, actual sampling and analysis procedures may be less than ideal with significant sampling time variations and analysis inconsistencies.

Optimization of sampling and analysis procedures can significantly improve dissolution test performance. Bath sampling can be performed serially (one bath at a time) or in parallel (all baths simultaneously) with the latter being the preferred option. Analysis is typically performed in a serial manner using either a direct sample analysis or a chromatographic separation for specific analyte detection. If multiple analytes are involved, multiple separations may be required. In addition to sampling and analysis optimization, integration of these processes into a single optimized sampling/analysis platform offers the advantage of accurate and reproducible control of all sampling and analysis operations without analyst interaction and the associated potential for error.

This poster will present current technology that can be applied to optimize automated dissolution testing. Options range from parallel bath sampling with multiple sampling probes and multiple flow injection valves for direct sample analysis to serial sampling with a single probe and multiple flow injection valves for automated analysis with multiple HPLC systems from a common sampling and analysis platform. The control options required to achieve these levels of automation will be presented.

### An open BenchMate™ system for the analysis of cleaning validation samples

*P. Deuringer and R. Schönneis, Bayer AG, Business Group Pharma, Quality Assurance, D-51368 Leverkusen, Germany*

Cleaning validations are necessary to verify that the pharmaceutical processing equipment is free of contamination from both active substances and cleaning agent after its use. The cleaning validation procedures for complex equipment results in a multitude of samples. The BenchMate™ system of Zymark® Corporation was found to be a suitable unit to analyse the large sample load.

In the QA laboratories, the swab samples which were taken from the equipment are directly transferred to the Zymark® BenchMate™ test tubes.

Some problems were encountered when using an 'off the shelf' commercially available BenchMate™:

- the cannula hit the swab sample in the tube while filling the sample tubes with solvent, so that the balance was influenced;
- the cannula became clogged with swap materials;
- sample solution was lost because the dipping speed of the cannula is very fast.

Therefore, the critical filling and sampling procedures were reprogrammed using the open-mode operation.

Additionally, the time-consuming manual input for multiple methods and the data transfer were optimized by connecting a PC. An Easyfill™ station was added to back-up samples.

### Automating a 'simple' moisture method

*Steven J. Salata, Nabisco, R&D, East Hanover, NJ, USA*

Nabisco's earliest venture into robotic laboratory automation started in 1986. The test chosen was moisture by vacuum oven weight loss, one of the most common in the food industry. It was considered 'simple' in the hands of any technicians, so improved productivity and precision was the goal. That system was developed, refined and used routinely over the course of the next 5 years. Constant tweaking and tuning by the chemist who was the 'owner' of the system allowed it to operate reliably enough to justify its space and maintenance cost, but disappointingly, it was not as 'good' as an experienced technician, even if its repertoire was limited to sample types that were homogeneous, easily pourable powders.

As our laboratory staff turned over, new users had no original developers around to coach them. Inconsistencies appeared in routine validation check samples without obvious cause. Increased concern with GLP and achieving higher productivity with fewer hands provided reason anew to redesign the system. This poster illustrates how that implementation led to identifying subtle but critical non-robotic factors and an unanticipated extra phase of the project to assure data quality.

### A robotic system designed for multiple applications

*Danh Nguygen and Martin Phillippi, The Clorox Company, Technical Center, Pleasanton, CA, USA*

Due to the relative high cost of robotics compared with other laboratory equipment and the high impetus in industry to regulate staff costs while increasing productivity, we have worked toward developing a robotic system capable of readily performing multiple applications. Although it is not unreasonable to utilize PyTechnology capability for interchanging stations or other table modifications, we have found that the per cent utilization drops dramatically when anything but minor set-up changes are required between applications. In addition, changing certain stations may present safety concerns due to their weight, size and table location presenting a whole new set of issues. Component switching between applications may also be a potential source of error if it is overlooked by the end user.

At our Research and Development Center, there is no single, redundant application that would justify the purchase of a robotic system if the criteria were that the purchase price be recouped in 1–2 years of service. In addition, changing and evolving product lines and project priorities cannot ensure the long-term utilization of an automated system based only on a single application. The table layout and programming of our Zymark XP robotic system were designed in such a way that multiple applications can be easily implemented on it by the end-user with minimal training. The types of applications,table modifications/adapters utilized, and peripheral equipment which enhance the system's versatility and multiple application capabilities will be discussed.

### Extraction and derivatization of acid and neutral impurities in illicit heroin using the XP robotics system

*Sam D. Cooper, Drug Enforcement Administration, Special Testing and Research Laboratory, McLean, VA, USA*

The instrumental phase being used in this laboratory for identifying acidic and neutral impurities in heroin samples requires approximately 60 min for an acceptable separation of all components. The manual method for extraction and derivatization of components required approximately 30 min. However, due to the instability of the derivatized components, this manual method, even with an autosampler, did not provide a workable solution to the problem of increasing productivity.

The XP robotics system enables the analyst to continuously prepare samples and proceed directly to the instrumental phase of the analysis.

In this presentation, the XP robotics system will be described with examples of programming which coordinates the extraction and derivatization procedures with the analytical processes. Photographs of different stages of the extraction procedures will be presented with accompanying descriptions of the advantages of this protocol.

### Automation of residue analytical methods for determination of pesticides in soil

*Herbert Sommer, Volker Koch and Markus Schuld, Bayer AG, Agriculture Center Monheim, FRG*

A new residue analytical method for determination of a new plant protection compound and its metabolites in soil has been developed. Different analytical techniques were used to improve precision, repeatability and reproducibility of methods for the determination of pesticides in soil and to reduce costs. For this, the most time-consuming steps have been automated or eliminated.

Three main modules are used:

- ASE 200 extractor
- Zymate XP robotic system
- API 365 or Quattro II—HPLC—MS/MS system

After extraction of soil samples with an ASE 200 extractor, the crude extracts are transferred to a Zymate XP laboratory robotics system that performs evaporation, reconstitution, centrifugation and filling into HPLC vials. If necessary, sample preparation is completed by a solid phase extraction station on the robotic system. Because of the high selectivity of HPLC–MS/MS, clean up is not currently used. The active ingredient and three metabolites are ionized using an electrospray interface. For quantification of the analytes, internal standards are added post-column utilizing a second injection port to compensate possible matrix effects in the ion source.

### Comparison of commonly used robots for laboratory automation

*Rodney Stockton, SLR Systems, Vancouver, WA, USA*

The most commonly used commercial robots utilized in laboratory automation systems will be presented. Important parameters, e.g. speed, reach, payload, communication and software descriptions will be compared between the various models. The presentation will include a purely physical description as well as more subjective comparisons based upon the author's experience.

Robots to be included (but certainly not limited to) in this comparison are manufactured by Beckman, Cavro, CRS Robotics, Fanuc, Hamilton, Hudson Robotics, Mitsubishi, Motoman, Panasonic, Seiko and Zymark. When multiple models from one manufacturer are available, all models normally utilized in laboratory operations will be covered.

### Formulation tools for cosmetic and personal care laboratories

*Stephen D. Phelan, Formation Systems, Westborough, MA, USA*

Over the past decade, there have been striking advances in computer hardware and software. Surprisingly little of this technology has trickled down to the R&D laboratories of household product manufacturers, which are distinguished by a much greater degree of variability than faced by discrete manufacturers. Recently, however, tools for both R&D and production chemists in process companies have become available. These tools address formula development and management across the enterprise, helping companies not only to improve the productivity of their chemists but also to achieve faster time to market. Such tools encompass marketing claims and specifications management; laboratory tests, methods and results; formula creation; guidelines and restrictions; formula approvals and distribution; production support; and manufacturing feedback. Use of these tools can drastically increase the value of the formula base, which is the primary intellectual property of the consumer goods manufacturer.

### Reaction screening and optimization by automated workstation-based systems: Part I

*L. C. Hsu, E. Webb and L. Pin, SmithKline Beecham Pharmaceuticals, King of Prussia, PA, USA*

The shortening of drug development timelines has caused Chemical Development to rethink its strategies for product development. One approach has been to incorporate robotics and automation into new drug development paradigms. This paper will discuss the hardware and the custom chromatographic configuration required for screening and optimization of the Suzuki coupling reaction. The reactions were performed in a STEM block and the samples were transferred to a Zymark BenchMate workstation for further sample preparation, including dilution and filtration. The treated samples were then analysed by an HPLC system that was directly connected to the BenchMate workstation. Finally the chromatographic system was configured so that data can be shared between Synthetic Chemistry and Environmental Research Laboratory via SmithKline Beecham network. This automated approach has the potential for accelerating drug development and process optimization in Chemical Development. The modification of BenchMate and the intranet data infrastructure will be discussed in Part II of the series.

### A custom Zymate system for printing and applying barcode labels

*Brent T. Butler and Joan Frezza, Department of Molecular Biochemistry, Glaxo Wellcome, Research Triangle Park, NC, USA; Joseph D. Simpkins and Anne Shrago, Department of Research Technologies, GlaxoWellcome, Research Triangle Park, NC, USA*

We have developed an automated plate labeller for labelling both 96- and 384-well assay target plates. This PyTechnology system employs a Zymate II robot with a Zymark storage carousel and a print and apply labeller. The labeller has been modified to house a barcode reader for plate verification. It takes about 1 min to get a plate, print and apply a barcode, and return the plate. The label contains both barcode and human-readable information. The labelled target plates are subsequently loaded on a compound handling robot [Frezza, J., Tomlinson, J. J., Klemp, M. and Butler, B. T., Fully automated compound distribution to multiple biochemical targets. *International Symposium on Laboratory Automation and Robotics*, Boston, MA, October 1997] for processing.

The system is controlled through a Visual Basic front end that retrieves barcode information from an ORACLE database. The barcode information is downloaded to an ASCII text file as a feature of the database software. A Visual Basic interface processes this information and transfers the appropriate data to EasyLab variables for use by the barcode labeller.

### The new era in high-throughput screening: miniaturized assays with 1536 Wells

*Günther Knebel, Greiner GmbH, Frickenhausen, Germany*

The necessity of miniaturized low-volume assay plates is mainly driven by limited availability of test material, savings in reagents, higher throughput and finally, less cost per well. Today, 96-well and 384-well plates are in use, but significant developments must evolve to meet further requirements to reduce cost per assay. This constraint leads directly toward even higher density plates.

In close cooperation, Bayer (Germany) and Greiner developed a new high-density plate on a non-proprietary basis that fulfills the requirements in fully automated systems. To utilize space most efficiently, the only focus was 1536 wells, a four-fold increase over a 384-well plate. The centre of a group of 16 wells is still unchanged at 9.0 mm, while the well-to-well pitch is 2.25 mm. A well volume of 12 μl allows bioassays and cell-based assays to be carried out. A new optimized well geometry in a square-rounded shape has been made to overcome wicking in the corners.

Furthermore, there has been strong evidence that clear bottom plates will be required. A new moulding and process technology enables us to manufacture unique clear bottom plates with ultra-thin films (75 μm). The film bottom offers excellent optical properties, reduces light tunnelling and minimizes well-to-well crosstalk. White opaque and black clear bottom microplates are advantageous when microscopic monitoring is required (e.g. cell-based assays). These clear bottom plates will have a major impact on assay application and new instrumentation for the detection of luminescence or fluorescence assays.

This talk will cover the application of solid and clear bottom plates in automated assays. Also, we will report on how liquid handling and imaging can be combined, and ongoing developments for updated 1536-well versions.

### Intranet control over several liquid handling work cells via active X™

*Jeffrey A. Guss, Bristol-Myers Squibb, Wallingford, CT, USA*

In an effort to streamline deployment and maintenance of liquid handling applications over several work cells, a novel approach using Active X™ was used to program a user interface coupled to vender-specific software. The benefits of undertaking this project are that it provides single-point application development, increased security and a thin operating environment. In addition, the application can be run 'off-line' from the work cells. This capability allows programming and debugging from remote locations. The Active X™ Intranet application contains an Access 97 database, interface tools for the liquid handling work cells, Hamilton Eclipse 4.1 software, liquid handling method selection, and a link to a paging system. The combination of the Active X™ Intranet application components and the consolidation of deployment to a distinct location makes tracking work cell activity and any errors a more manageable task.

### Fluorescence-based assays in microwell plate format for high-throughput drug screening

*Steven F. Gessert, Suzanne W. Jones, A. Lee Lang, Toddy Sewell and Sydney B. Shaw, Chromagen, San Diego, CA, USA*

Rapid advances in the generation of potential drug targets through genomics and concurrent strides in the creation of drug candidates through combinatorial chemistry have dramatically increased the demand for sensitive, rapid, automatable drug screening assay formats. Chromagen has developed fluorescence-based gene transcription and protease assays in a high-throughput-compatible microwell plate format. These assays incorporate three of Chromagen's proprietary core technologies:

(1) novel fluorophores and fluorogenic enzyme substrates specifically developed for simultaneous multianalyte detection;
(2) microwell-based, solid-phase extraction chemistries for the isolation and purification of nucleic acids and peptides; and
(3) a scanning photon-counting spectrofluorometer capable of providing simultaneous multi-target detection.

The gene transcription assays [developed for the human cytokines tumour necrosis factor-alpha (TNF-α and interleukin-6 (IL-6)] utilize DNA probes designed to recognize specific sequences in target mRNA, and require neither nucleotide amplification nor reporter gene constructs. Detection levels in the attomole range (10E-18 mole) of specific mRNA are routinely achieved using lysates from 10E5 cells per assay well. The mRNA assay platform is applicable to numerous genes and can be optimized for cell lines for a variety of organisms. The protease assays developed for the coagulation cascade are homogenous assays and measure kinetics of substrate metabolism.

### POLARA: a versatile architecture for scheduling and running microplate assays

*Daniel Sipes, Joyce Kuoha and Jonathan Rosen, Ligand Pharmaceuticals, San Diego, CA, USA*

POLARA (Programming Architecture for Laboratory Automation) is a Windows NT-based software package for laboratory automation produced by CRS Robotics (Burlington, Ontario, Canada). An overview of the installation and operation of POLARA on an existing custom CRS Robotics system for cell-based assays in microplates will be presented.

### NMR and automated reaction monitoring

*M. Armitage, J. Richards, K. Sutcliffe and S. Moore, SmithKline Beecham Pharmaceuticals, Tonbridge, Kent, UK*

Chemical Development within SmithKline Beecham is charged with developing an efficient process for the manufacture of drug substance. Such a process must be demonstrated to be safe, robust, economically viable and environment friendly at a manufacturing scale. A thorough understanding of the chemistry using reaction monitoring can help achieve these goals.

Monitoring has traditionally been performed off-line using techniques, e.g. TLC or HPLC, but NMR potentially gives much more information about the pathway of a given reaction. An on-line NMR reaction monitoring system therefore would be beneficial to the scale-up process. Whilst proprietary systems specifically designed to perform these tasks are becoming available, we tried to implement such a system with what was available to us. This poster describes our experience with a reaction monitoring set-up using an NMR spectrometer fitted with an LC-NMR probe and a Gilson 231 autosampler.

The Gilson 231 autosampler consists of an XYZ robotic arm and a diluter unit. The instrument is programmable and capable of sampling, dilution and injection of aliquots of sample, and should therefore provide a suitable system for introduction of a sample into the NMR spectrometer. Our goal was to automate the system as far as possible such that unattended operation could be achieved.

### Automated liquid handling confirmation of a Tecan Genesis

*Jimmy Bruner, Rusty Czerwinski and James Ormand, Glaxo Welcome, Research Triangle Park, NC, USA*

An application has been developed to perform automated gravimetric confirmation of the Tecan Genesis's liquid handling system. The application was developed in response to the time-consuming manual liquid handling confirmation required for Good Laboratory Practice (GLP) compliance. The system consists of a Tecan Genesis 100, a Sartorius RC250S analytical balance, and a custom ActiveX DLL for communicating with Tecan Logic version 2.11. The system components were integrated using Microsoft Excel 97.

First, an ActiveX DLL was created to allow communications between external applications and Tecan Logic version 2.11. The LogicPipeComm.DLL consist of two components, connection and communications. The connection component was created in Microsoft Visual C++ and connects to the Logic pipe. The communications component was created in Microsoft Visual Basic version 5.0 to access the connection component for a pipe handle, and to write data to and from the Logic pipe.

Second, an Excel 97 VBA (Visual Basic application) was created. The Excel application integrates the LogicPipeComm.DLL, Sartorius RC250S analytical balance, and Tecan Logic version 2.11. The application controls all balance operations, starts the Tecan calibration method, receives data from the balance, and calculates the mean, standard deviation and per cent bias of replicate aliquots.

The LogicPipeComm.DLL gives us the capability to control Tecan Logic via external programming platforms, e.g. Visual Basic and Excel. Integration of the Tecan and a balance reduces the time the scientist must spend recording weight data needed for GLP compliance.

### Versatile object weighing on a Zymark BenchMate

*Jimmy Bruner, David Igo and Sonia Shamdasani, Glaxo Wellcome, Research Triangle Park, NC, USA*

An application has been developed to perform fully automated weighing of various objects on the Zymark BenchMate. The application, known as 'ZyWeighing', was developed in response to the time-consuming manual method of weighing containers and contents used in chemical investigations. The ZyWeighing system consists of a standard Zymark BenchMate, Zymark EasyServ OLE Server, custom object carriers, and custom programs.

The software consists of three elements. First, a Visual Basic program was developed using OLE technology to access the EasyServ OLE and Microsoft Excel. Second, an Excel workbook was created for capturing the experiment and weight data. Finally, EasyLab programs were created for running on the BenchMate Controller.

Customer carries were developed for objects of various size. Each carrier was designed to fit the standard BenchMate rack.

The system has been shown to reduce the amount of time a scientist spends performing weighing operations for 4 h to 30 min for a standard set of 100 samples. Precision was assessed by weighing 50 individual samples 100 times using the resident Sartorius Model XH110 balance. The standard deviation was less than or equal to 0.1 mg. Accuracy was examined using standards having masses ranging from 5 mg to 2 g. The weights ranged from 100.00 to 101.00% of their theoretical values.

### Ultra-high-throughput data analysis

*Neil Carlson, Nick Ware and Michelle Palmer, Assay Development Services, Tropix, Bedford, MA, USA*

Now that it is possible to run 100 000 assays in a 24-h period, it is critical to look beyond the physical issues of running these assays, and to address the impact of ultra-high-throughput screening (UHTS) on post-assay data processing. Data collection, storage, back-up and protection are only a small part of the overall problem. The more challenging task involves presenting this large volume of data in such a way as to allow scientists to make correct, timely decisions about potential errors in plate processing or treatment during the assay; to mark specific compounds, rows or entire plates for retesting in a particular assay; to quickly find statistically significant 'hits'; and to assign a compound for further testing in subsequent assays. This poster presents one approach at addressing these issues, and explores future possibilities with regards to data presentation and 'pre-analysis' of the data generated during UHTS.

### Strategies for high-throughput HPLC purification for combinatorial chemistry

*J. Carmody, U. Neue, R. Crowley and C. Andres, Waters Corporation, Milford, MA, USA*

Traditionally, the search for biologically active molecules has involved the synthesis of one-molecule-at-a-time discovery strategies. This has proven to be a very time-consuming and labour-intensive process for the discovery of new drugs, catalysts and materials. New combinatorial chemistry techniques have reduced synthesis times by allowing simultaneous generation of a large number of chemical variants, several of which may be active leads. Once this lead generation process is complete, the resultant combinatorial library is subjected to high-throughput screening techniques, one of which is HPLC. After biological activity testing, there is still the need to isolate the active molecules without creating a bottleneck in the laboratory.

Lead optimization, for many compounds, requires that only a few micrograms or milligrams of a compound be purified to confirm the preliminary results. In this paper, we will show straightforward HPLC strategies to developing fast gradient methods to quickly scale-up and purify target compounds from other inactive combinations

### Strategies for high-throughput HPLC analysis for combinatorial chemistry

*J. Carmody, U. Neue, R. Crowley and C. Andrews, Waters Corporation, Milford, MA, USA*

Traditionally, the search for biologically active molecules has involved the synthesis of one-molecule-at-a-time discovery strategies. This has proven to be a very time-consuming and labour-intensive process for the discovery of new drugs, catalysts and materials. New combinatorial chemistry techniques have reduced synthesis times by allowing simultaneous generation of a large number of chemical variants, several of which may be active leads. Once this lead generation process is complete, the resultant combinatorial library is subjected to high-throughput screening techniques, one of which is HPLC. Due to the plethora of compounds generated by the combinatorial chemistry technique, minimizing analysis time is paramount to meeting the major challenge of isolating the desired compound from other indigenous material as quickly as possible. Optimization of the HPLC screening process to achieve shorter analysis times is not always intrinsically straightforward. In order to achieve compressed analysis times, there is a need to more completely understand the effect that the column characteristics (i.e. particle size and column length) and the operational parameters (i.e. flow rate and gradient time) have on the selectivity and resolution of the separation; the resolution and the selectively being the two characteristics of the separation most affected by changes in the column and operational parameters.

In this paper, we will show straightforward HPLC strategies to developing fast gradient methods to quickly resolve target compounds from other inactive combinations.

### Sample dissolution and replication station for plates from combinatorial chemistry

*Gerhard Mihm, Department of Chemical Research, Screening Support, Boehringer Ingelheim Pharma KG, Biberach, Germany*

As a result of the rapidly increasing number of compounds prepared in the combinatorial chemistry groups, there is an urgent need in the chemical repositories to optimize the processing of the samples for high-throughput screening (HTS). Samples have to be dissolved and aliquoted into several plates and tubes in order to transfer them to the HTS groups. In addition, an efficient and reliable tracking of the platemaps is mandatory. We have designed and implemented, together with Zymark UK and Zymark Germany, a workstation-type robotic system based on the Zymark RapidPlate, including a robotic arm to handle the plates. Plates are tracked by barcode labels. Data management is via file transfer between the workstation and the dispensary ORACLE database.

### Efficient handling of compound solutions for HTS and follow-up assays using a new robotic system

*Gerhard Mihm, Department of Chemical Research, Screening Support Unit, and Rudolf Haider, IS Department, Research Support Team, Boehringer Ingelheim Pharma KG Biberach, Birkendorferstrasse 65, Germany*

The demands for rapid and reliable handling of compound solutions for high-throughput screening activities and follow-up assays are increasing steadily. In addition, the numbers to be stored and manipulated in the compound dispensaries are also increasing due to the activities in combinatorial chemistry.

In order to meet those needs, we have implemented an automated storage and retrieval system for compound solutions in microtubes, microtubes in the corresponding racks and microtitre plates (REMPASS). The samples are stored in special storage racks at 4 °C and manipulated with a 14axes robotic system. The coordinates of the samples are kept in a dedicated database. Requests for compounds are processed via the database used in the compound repository for the administration of all samples. The robotic system used for the manipulation of the samples is presented here as well as the interface to the dispensary database.

### Does your liquid handler pipette accurately?

*James Ormand, Jimmy Bruner, Sarva Tadepalli and Cole Harris, Glaxo Wellcome, Research Triangle Park, NC, USA*

Many bioassays require organic solvents to solubilize high concentrations of compounds, precipitate proteins, quench enzymatic reaction, etc. Manufacturers, e.g. Tecan, Packard and Beckman have designed a variety of liquid handlers to automate these bioassays. Water is the benchmark liquid used when evaluating the accuracy and precision of liquid handling equipment. But, how accurately does this equipment handle non-aqueous liquids?

In this example, preparation of plasma standards from acetonitrile stock solutions using a Tecan Genesis 150 with Logic version 1.85 revealed a problem with the accuracy of delivering organic solvents within the normal volume range of the instrument. The observed concentrations of standards prepared using the automated technique were found to be ~15% lower across the range when compared to manually prepared standards for two separate runs. Gravimetric measurements revealed that he system was only delivering ~ 84% of the requested volume of acetonitrile, and similar results were observed with methanol. The solution to this problem was found in the recently released Tecan Logic version 2.11 through the use of liquid classes.

This poster will discuss the challenges associated with handling non-aqueous liquids and how manufacturers, e.g. Tecan and Packard handle these issues.

### A flexible, generic robotic system that performs protein precipitation assay for research support

*J. A. Short, E. D. Lynch and T. L. Lloyd, DuPont Pharmaceuticals Company, Newark, DE, USA*

As a drug discovery program begins to narrow the search for a specific compound to advance into drug development, the final candidate compounds within that research class are studied *in vivo* in different animal populations. Research support bioanalysis involves these first *in vivo* sample analyses of a variety of closely related compounds. These studies are characterized by small sample batches typically 20–80 samples per compound, where analysis of multiple compounds is often required within a single analytical run. There are lots of studies for compounds closely related in structure, therefore leading to the generation of a large number of different standard curves for quantitation. Sample matrices vary considerably and sample volumes are often small (200 µl). Because these are the first *in vivo* studies with these compounds, analysts are forced to guess rather broadly at sample concentrations.

The investment in method development at this stage is typically measured in hours, with almost all of the research support work involving protein precipitation for sample preparation. The scientists performing these studies typically have broad responsibilities, with the bioanalytical component only comprising a small portion of their duties. As such, an automated platform for these applications must have a friendly user interface such that users can walk up and quickly run the system with minimal training or understanding of the automation. The system must have a high degree of flexibility to accommodate the method changes associated with each set of samples between or within analytical runs.

Such a system has been created and implemented. Features of this system will be presented. Some of the more formidable challenges to this approach, encountered in working with the Factor Xa class of compounds, will be explored and discussed.

### Automated HTS monitoring technology: laser fluorescence methodology for fingerprinting food, beverage and pharmaceutical products

*R. P. Gill, W. Smith and R. H. Selinfreund, Lion Laboratories, Saybrook, CT, USA*

The absorption of light by matter is related to molecular structure. Some molecules can momentarily store energy by the excitation of electrons from the ground state to excited singlet states. Relaxation from an excited singlet state results in fluorescence via the emission of light at a different wavelength. The interaction of fluorescent dyes with complex mixtures, e.g. foods and beverages, is highly reproducible, quantifiable, and can be used as a particularly valuable method to characterize mixtures.

Using a proprietary library of dyes and the resulting interaction(s) with single or multiple analytes, Lion Laboratories has developed a method to numerically describe fluorescent fields for foods and beverages. This process in the most simple terms can be thought of as mapping the fluorescent field of an analyte or mixture of analytes via interaction with specific dyes or dye combinations. The dye analyte interactions may involve complexation and/or the formation of ionic or covalent bonds. The fluorescence utilized can be in the ultraviolet, infrared or visible regions of the electromagnetic spectrum, and is expressed in a digital fashion to develop a multiple fluorescent profile of a mixture designated as a fingerprint. In certain cases, this can be used as a specific assay for a single analyte that may be either a key ingredient or an undesirable contaminant; or more generally, the fingerprint can be used to differentiate authentic from non-authentic samples.

Lion has developed this process utilizing 96-well plates, robotics and automated dilution/sampling techniques to provide a technology (patented) to assess authenticity of complex mixtures. Applications include spirits, beverages, fruit juices, flavours, natural extracts, e.g. vanilla, as well as individual ingredients, e.g. caffeine. The technology is also of value to characterize or fingerprint chemical processes, and it allows a simple mechanism for protection of intellectual property. The poster includes an overview of test design, robotic HTS infrastructure, sample handling and data management/presentation.

### Microadsorptive tips for sample preparation prior to mass spectrometry

*Rich Garretson and Donald G. Sheer, Millipore Corporation, Bedford, MA, USA; Melanie Lin, Ken Parker and Martin Hornshaw, PerSeptive Biosystems, Framingham, MA, USA*

The ability to remove salt and detergent from protein or peptide samples at the nanogram level has become rate-limiting in structural characterization studies. In many cases, the high sensitivity afforded by mass spectrometry is compromised by interfering contaminants that suppress fragment ionization and obscure mass peaks. This complication along with the difficulties associated with microlitre volume and nanogram mass handling makes sample preparation a significant challenge. To address these demands, an adsorptive pipette tip containing previously evaluated reverse phase and ion exchange resins [*J. Biomolecular Techniques* (1997), **1**, 0001, 0009] is presented for direct mass spectrometry. Sample recovery, fidelity and reproducibility were demonstrated using HPLC and MALDI. The ability of these devices to remove salt and detergents enabled the acquisition of excellent MALDI spectra for a variety of controlled digests.

### Use of the Cytofluor 4000 microplate fluorimeter and twister plate loading robot for high-throughput yeast viability screens

*Christopher L. Haber and James C. McGuire, Discovery Technologies, Pharmacia & Upjohn, Kalamazoo, MI, USA*

Fluorescent dyes have been used extensively as indicators of cell viability. Because of their robustness and ease of use, they are easily incorporated into high-throughput, whole cell screens with cell viability end-points. We have routinely used the alarmarBlue redox indicator in such screens and the Cytofluor 4000 microplate fluorimeter for determining the fluorescence intensity of the samples. Recently, a robotic plate handling arm (the Twister) has become available for the Cytofluor 4000. Here we describe the performance of the Twister-outfitted Cytofluor in screening for least viability using 96-well and 384-well plates. The Twister currently holds up to 20 plates. The software allows multiple plate data in a single file, and can accommodate $>20$ plates per file (sequential runs of up to 20 plates each). Raw data are exported in a variety of file formats for use in data processing programs. Throughput with 96-well plates with the Twister was 45 plates/h, 15 plates/h with 384-well plates. This system was used to screen at 116 000 compound library against a *Saccharomyces cerevisiae* permeability mutant.

### High-performance automated chemistry: synthesis and beyond

*Richard Gray and Clare Ruddick, The Technology Partnership, Melbourn, UK*

This poster illustrates various key features of the Myriad Core System (MCS) and Personal Synthesizer (PS) which were developed at The Technology Partnership (TTP) in collaboration with seven leading pharmaceutical companies. In particular, it will emphasize the most recent developments which expand the range of chemistry possible, including reactions with reactive gases at up to 4 bar pressure and reactions involving distillation steps. The new automated liquid–liquid extraction module is also described. This poster addresses the most common solution phase work-up procedure in a convenient and efficient instrument.

### The automated synthesis of a solid phase Ugi library

*George C. Morton, Timothy F. Herpin, Joseph M. Salvino, Jonathan S. Mason, Sheng-Yuh Tang, Gerald McGeehan and Richard Labaudienier, Lead Discovery, Rhone-Poulenc Rorer Central Research, Collegeville, PA, USA*

A novel resin-bound isonitrile was used in the Ugi multicomponent reaction to generate a library of dipeptoid carboxylic acids. One thousand, one hundred and thirty-six compounds were synthesized on two ACT 496 MOS robotic synthesizers. A Tecan RSP 5051 liquid handling robot was used to transfer a slurry of the resin into the ACT reaction blocks and also to transfer the final products. The automation protocol will be presented along with the general reaction scheme.

### Managing combinatorial chemistry analytical data using an electronic notebook interface and open database technology

*Tom Clemow, Howard I. Cannon and Curtis Marx, Groton NeoChem, Acton, MA, USA*

Improvements in combinatorial organic synthesis have made producing thousands or tens of thousands of compounds per month practical. Today's HPLC, MS and NMR instruments accommodate quantitative or qualitative analysis of thousands of samples per month with a limited number of instruments.

Existing data management tools, however, cannot easily or automatically combine data from multiple analytical techniques, or with structural, activity and compound location data. Without suitable management software, analytical data are disconnected from other drug discovery data. Future improvements in synthetic, analytical and screening automation will exacerbate this bottleneck in drug discovery.

The NeoLink/cc software automatically imports chromatographic, MS and NMR results into ORACLE or any ODBC compliant database. This database also contains links to other drug discovery data including chemical structure, compound location and activity. The software interface includes powerful data query and data display functionality. The notebook interface provides a common viewer for raw and procesed data from different analytical techniques. Once data are associated in a notebook format, they can be saved and forwarded to other researchers to support rehearsal or production syntheses. Data extraction and association can be automated using data templates for routine data analysis and reporting. This software tool effectively links analytical QC data to chemical library and HTS databases for more comprehensive and timely access to chemical synthesis information.

### A powerful new screening technology utilizing capillary electrophoresis

*Y. M. Dunayevskiy, L. G. Rashkovetsky and D. E. Hughes, Cetek Corporation, Marlborough, MA, USA*

A new screening technology based on laser-induced fluorescence with high-resolution capillary electrophoresis (CE) has recently been developed. This technology greatly enhanced the ability to discover valuable 'hits' in complex mixtures, e.g. natural samples (crude extracts, broth), combinatorial chemistry libraries and combinatorial biology mixtures. Examples using several targets and discriminating between strong, moderate and weak binding hits will be shown. With natural samples, these very sensitive CE assays can follow active fractions through multiple purification steps, and typically find numerous hits per active sample.

### A high-throughput luciferase gene reporter assay system: LucLite™, LumiCount® microplate luminometer and Twister™ universal microplate handler

*Li Li and Steven Lane, Packard Instrument Company, Meriden, CT, USA*

Reporter gene assays have been widely used in studying regulation of gene expression. The bioluminescent luciferase reporter assay has become the method of choice in reporter gene studies, especially in many drug-screening applications, because it is the most sensitive, simple and rapid assay for quantitating transcriptional activity. In this paper, we present a simple procedure or automating a luciferase reporter gene assay using the LucLite™ luciferase reporter system, the LumiCount® microplate luminometer and the Twister™ universal microplate handler. The LucLite reagent kit provides true long-lived glow luminescence signal for detection and a simple homogeneous assay procedure. Tle LumiCount microplate luminometer enhances these advantages with a high-performance detection design, allowing detection of firefly luciferase at the attomole concentration level in both 96- and 384-well microplate formats. The signal is linear over an extended enzyme concentration range of five orders of magnitude using one instrument setting. The same attomole level sensitivity and extended linear detection range are achieved using the Twister universal microplate handler, allowing for an increased sample throughput and walk-away convenience. The resulting performance in sensitivity, throughput, accuracy and cost advantages makes this combination of reagents, instrument and plate stacker an ideal system for microplate-based gene regulation studies and high-throughput drug-screening applications.

### New techniques in solid-phase synthesis: gaseous chemistry for parallel array synthesis

*Thomas J. Baiga, Charybdis Technologies, Carlsbad, CA, USA; Gregory P. Roth, Boehringer Ingelheim Pharmaceuticals, Ridgefield, CT, USA*

As part of an on-going program to further develop the versatility of the Calypso Reaction Block System,

Charybdis teamed up with Boehringer Ingelheim to advance the state-of-the-art solid-phase synthesis paradigm. These new techniques involved gaseous chemistries, where active synthesis reagents were delivered to the Reaction Block as gases using the Calypso Gas Manifold. Three new chemical methodologies were developed: Pd-catalysed aromatic nitro group reaction, Pd-catalysed hydrogenolysis as a Wang resin linker cleavage protocol, and Pd-catalysed Still-type carbonyl insertion.

These chemistries were originally developed manually in the Calypso Reaction Blocks. But to further enhance and explore this methods development program, several automation platforms have been integrated, notably the Iliad PS2 Personal Synthesis System, the Calypso 4X Shaker Workstation and the RapidPlate® multichannel pipetter. The RapidPlate represents the newest product in an extremely versatile line of workstations to be integrated with the Calypso System.

### Throughput organic synthesis for discovery library production: the automation of the Ugi multi-component condensation reaction

*Traci Trotman, Charybdis Technologies, Carlsbad, CA, USA; Chris Hulme, Rhone-Poulenc Rorer, Collegeville, PA, USA*

Faced with the daunting task of automating the synthesis of more than 3000 compounds per week, scientists of Rhone-Poulenc Rorer have integrated several technologies into a unique combinatorial chemistry production facility. Utilizing the Ugi multi-component condensation reaction as a probe for molecular diversity in their discovery libraries, the RPR Team has successfully automated most solid-phase synthesis operations.

Charybdis Technologies Calypso Reaction Blocks (96 well, filtration variant) were initially prepared with synthesis resin. This task was automated by integrating the Calypso RM onto a RapidPlate® 96 locator plate, and simultaneously dispensing resin materials as an isopycnic slurry. The Calypso RBs were drained, washed and prepared for synthesis on the Iliad PS2 personal synthesis system. The Iliad delivered reaction solvents and diversity reagents to the 96-well reaction blocks. Reaction incubation was performed off-line to maximize the compound throughput for the task-specific workstation network.

### Use of the nQUAD technology for nanolitre dispensing in microplate applications

*Dennis Stachelek, Don Rose, Tony Lemmo and Tom Tisone, Cartesian Technologies, Irvine, CA, USA*

Cartesian Technologies has developed a nanolitre, quantitative aspirate and dispense (nQUAD) technology for use in microplate applications. The technology couples the precision displacement resolution of a stepper motor-driven syringe pump with the rapid actuation capability of a micro-solenoid valve. The result is a dispenser technology with the following features:

- quantitative dispensing of nanolitre volumes (down to 4 nanolitres);
- dispense in a non-contact mode for easier mechanical alignment;
- rapid dispensing speed; and
- wide dynamic dispense volume range (low nanolitres to mid microlitres).

Increasingly, there is interest in using high-density microplates (384 wells and greater) for screening applications to achieve higher throughput and reduce plastic and reagent costs. Because these microplates have a well volume as small as 2 μl, the typical dispense volume is in the sub-microlitre or nanolitre range. With its ability to quantitatively dispense in this volume range, the nQUAD technology has found recent application as a useful technology in microplate applications. This poster will show the mechanism, characteristics and practical issues in applying the nQUAD technology to microplate screening applications.

### Integration of a 384-well plate washer with a robotic plate handler

*Jim Breunig, Mitotix, Cambridge, MA, USA*

The advantages of the 384-well plate format for high-throughput screening can be greatly enhanced with the capability to automate the delivery and removal of the plates to the instrument. A recent integration of a Skatron Instruments, EMBIA 384-well plate washer with a Zymark Corporation Twister™ universal microplate handler was tested and evaluated. Results of ease of set-up, ease of use, throughput, reliability and overall suitability for the high-throughput laboratory are presented and discussed.

### Multifunctional robotics compatible washer for high-throughput screening

*Paul Held and Gary Barush, Bio-Tek Instruments, Winooski, VT, USA*

Today's research requires that instrumentation be cost effective, reliable and easy to use. Bio-Tek Instruments would like to introduce the $El_x405$ multifunctional washer. Because today's assays require multiple washing formats, the $El_x405$ can be configured in three different modes of operation: (i) a combination of 96-well or 384-well washer manifold; (ii) a 96-well only manifold; and (iii) a 96-well version with magnetic bead capabilities. The 96/384 manifold has a unique design (patent pending) which provides for independent control of aspirate and dispense tube location and height, enabling bubble-free fluid-dispense and overflow protection in 384-well plates. The 96-well manifold allows for overflow protection, as well as 'bottom washing', and standard features have been included to make the $El_x405$ washer robotics friendly and ideal for automation. Features, e.g. vacuum detection, are provided to ensure that the vacuum pump is switched on and vacuum vessel are connected. Flow protection alerts the user to an interruption of flow to the dispensing manifold. The washer has a waste detection device, which through software prevents overflow of waste into the vacuum pump. Because buffer is drawn from reservoirs by suction from a syringe pump, pressurized buffer containers are not required for operation. The optional buffer switching module allows for the automatic switching from up to four different buffers. The washer also has a separate priming trough to allow for automatic priming ('autoprime') and automatic rinsing ('autorinse') during wash procedures. The $El_x405$ is robotics friendly with RS232 connections that allow communication with several robotic devices. Assay protocols are capable of being downloaded from a separate PC or programmed through a washer keypad. Besides being versatile, the washer is lightweight (< 13.5 kg), compact (41 × 41 × 26 cm) and capable of 110 and 220 V operation.

### An automation-friendly high-performance and high-throughput (HTS) assay detection system integrated with a high-capacity microplate handler

*Amer El-Hage, Carl Wright, Mike Biros and Doug Modlin, LJL BioSystems, Sunnyvale, CA, USA*

An overview of the design, performance and features for laboratory integration of the Analyst™ will be presented. Analyst™ is LJL BioSystems' new multi-mode detection platform for high-throughput screening (HTS) for accelerated drug discovery. The instrument was designed from conception to operate in an automated laboratory environment as part of a workstation or robotics line set-up. Analyst™ has already been successfully installed and integrated into several automated laboratories with robotics systems and workstations with minimal effort. An integrated microplate stacker option to the instrument improves throughput and efficiency, and a robot-friendly microplate carrier reduces both set-up time and process errors. In addition, a graphical user interface that runs on a local host in Windows 95 or NT facilitates system set-up and method development. We will highlight in this poster the integration of the Analyst with the Zymark Twister™ microplate handler as a specific example.

### Test liquid handling systems from 4 μl to 325 μl quickly and easily with PathCheck™ sensor

*Evelyn McGown, Kirk Schroeder and Dean Hafemen, Molecular Devices Corporation, Sunnyvale, CA, USA*

As part of a laboratory quality system, pipettors and dispensers should be checked regularly to verify performance. Gravimetric methods for pipettor calibration are adequate for single-channel devices but are impractical for multichannel pipettors. Spectophotometric methods typically require an added dye, which is convenient and potentially erroneous because the dispense volume of the liquid may be affected by the dye. The Molecular Devices SPECTRAmax® Microplate Spectrophotometers employ a patented PathCheck sensor which offers a fast, reliable method for checking performance of multichannel pipettors and dispensing stations using dispense reagent, without added dye.

The performance test is performed by dispensing the reagent into a 96-well microplate and measuring the optical pathlength in each well. Precision is calculated directly from the pathlength data and accuracy is determined by reference to a pathlength/volume standard curve. Dispense volumes between 30 μl and 325 μl can be tested directly with typical standard deviations of 0.003 cm (or 0.5 μl in a half-area well microplate). A difference method enables volumes from 4 μl to 30 μl to be accurately tested with a standard deviation of 0.015 mm. The method is fast, accurate and ideally suited to test automated liquid handling stations, especially 96-channel pipettors.

### A novel fluorescent HTS platform using homogeneous miniaturized cell- and bead-baseds assays for drug discovery and development

*Elana Swartzman, Sheri Miraglia, Lolita Evangelista, Kenton L. Lohman and Pau Yuan, PE Biosystems, Foster City, CA, USA, David M. Heffelfinger, Ed Goldbery and Bala S. Manian, Biometric Imaging, Mountain View, CA, USA*

Detecting cellular biochemical or molecular events in reaction to exogenous drug stimuli in live cells has always been a challenge to drug development groups. Homogeneous or 'no wash' assays on live cells have been especially difficult and have lacked the sensitivity needed for effective evaluation of biological events. A number of instrument and biochemical technologies have been developed to address this issue. Most have concentrated on elucidating drug effects on live cells earlier in the drug screening process. In most cases, however, high-throughput formats have been sacrificed for the increase in information.

In a co-development effort, PE Biosystems and Biometric Imaging have developed a new, two-colour, fluorescence-based instrumentation which enables the user to rapidly screen large numbers of drug candidates on live cells using homogeneous assay formats in standard microplate formats up to 1536 wells. The instrument is based on the laser scanning microvolume fluorometry technology developed by Biometric Imaging for cytometric analyses.

Homogeneous receptor/ligand studies, signal transduction mechanisms and cell death determinations can all be made rapidly on live cells. Data are collected for each individual cell in the scan area, which is unique for high-throughput instrumentation. The two-colour format provides the user with the ability to evaluate two cellular biological events simultaneously in a single cell in a high-throughput mode.

In addition, two-colour homogeneous, bead-based versions of standard biochemical assays previously developed for solid phase technologies, e.g. immunoassays, are easily accomplished on this platform.

We will describe the specific, homogeneous, biochemical systems and instrumentation that we have developed to investigate the effect of candidate drugs on live cells in high-throughput formats.

### Detecting the binding of fluorescent neuropeptide Y to CHO-K1 cells expressing NPY receptor subtypes using fluorometric microvolume assay technology (FMAT) in a high-throughput, cell-based format

*Sheri Miraglia, Elana Swartzman, Lolita Evangelista, Kenton Lohman and Pau Yuan, Perkin Elmer Biosystems, Foster City, CA, USA; Bala Manian, Biometric Imaging, Mountain View, CA, USA*

The development of molecules that mimic, enhance and/or block the interaction of bioactive peptides with cell surface receptors, in particular the subset represented by the 7-TM (transmembrane) G-protein-coupled receptors, is a major focus of pharmaceutical development. The rapid identification of potential leads is a crucial first step in this process and would be greatly facilitated by simple, homogeneous, high-throughput screening assays. Currently, the scintillation proximity assay provides a homogeneous format for monitoring receptor–ligand interactions; however, the assays are all bead-based and require the use of radioactivity. We have developed a technology, fluorometric microvolume assay technology (FMAT), which enables detection in the red region and is compatible with various formats, including 96-, 384- and 1536-well plates. Using this technology, we are able to detect the binding of fluorescent neuropeptide Y to transfected cells expressing either the neuropeptide Y type 1 receptor or type 2 receptor, and identify the IC-50 values of competing unlabelled peptide. Ligand binding assays were performed in high-density, low-volume 384-well plates. This technology can be used in a cell- or bead-based format, and is unique in that it is simple, homogeneous, high throughput and non-radioactive.

### Performance of Labsystems' microplate instrument interfaced with the Assist microplate handling device

*Ron Gulka, Labsystems, Franklin, MA, USA*

The Labsystems' Assist microplate handling device easily and economically automates the manipulation of 96- and 384-well plates. The Assist can be interfaced to a wide range of Labsystems' microplate instrumentation, including dispensers, washers and readers. Highlighted will be the performance specifications of the Assist and the Multidrop 384 dispenser, Wellwash Ascent plate washer, Multiskan Ascent photometric reader, Fluoroskan Ascent fluorometric reader, and Fluoroskan FL combination fluorometric and luminometric reader.

### A versatile, fully automated open-access work-up station designed to serve the needs of the rapidly expanding field of multiple parallel synthesis (MPS)

*Gordon A. Hamlin, Zeneca Pharmaceuticals, Alderley Park, Macclesfield, Cheshire, UK*

The rapid growth of multiple parallel synthesis (MPS) in our laboratories has created a demand for a robust, easily accessed automated work-up procedure, as the manual work-up of large numbers of small-scale reactions is both time consuming and tedious. Manual work-up is also a rate-limiting step in the generation of large numbers of compounds for test.

Work-up in chemical organic synthesis consists of a series of post-reaction operations designed using differential chemical properties to remove excess reagent or starting material, reagent products and, where possible, reaction by-products. Careful consideration of post-reaction operations as a clean-up step can obviate the requirement for purification. This has been an attractive feature of some MPS at Alderley Park, which we have attempted to address with automated procedures.

Generally, work-up can be resolved into four operations: filtration, solvent addition (dilution, trituration), washing and separation (partition). The ordering and selection of these four basic operations through an operator-controlled interface will, therefore, create a generic work-up program. This presentation will describe the flexibility and power of a Zymate XP-based generic work-up protocol as well as the safeguards necessary for its smooth operation as an open-access facility. Thought is also given to the development of a condensed, bench-top system for use in a standard chemistry laboratory fume cupboard.

### Solid phase extraction as an efficient method of purification for automated synthesis

*Marian Young, Harold Weller, Jacques Roberge and Walter Ruediger, Bristol-Myers Squibb Pharmaceutical Research Institute, Lawrenceville, NJ, USA*

As pharmaceutical companies continue to search for new drugs, automated synthesis is becoming more widely used. These synthetic procedures often produce compounds that need purification before they are submitted for biological screening.

Solid phase extraction (SPE) is an automated high-throughput purification method that was originally used for concentration of biological and environmental samples. We have modified these methods to be useful for purification of synthetic compounds. Most commercial instruments using SPE cartridges process one sample at a time. Because each sample can take 20–60 min for purification, a modest automated run of 96 compounds can take several days to purify.

We have developed with Bohdan an instrument that is capable of processing a set of 96 compounds purifying eight samples at a time. This instrument has the versatility of collecting up to three fractions for each sample, and using either 3 or 6 ml cartridges. This capability has allowed the purification of 288 compounds in a single day. We designed the graphical interface to be very user friendly, as we encourage walk-up use of our automated equipment. The development of instrumentation, modification of SPE resins and purification methods used to make SPE a useful and efficient method of purifying libraries of compounds will be discussed.

### Automation and library production at Selectide

*Eric Wegrzyniak, Kirsten Bjergarten, Marty Vanderstelt and Daniel Schirlin, Selectide, a subsidiary of Hoechst Marion Roussel, Tucson, AZ, USA*

In order to complement the well-known 'one bead one compound' or 'split and mix' technology originally developed by Selectide, we have designed a system for high-speed synthesis (HSS) of combinatorial libraries in commercially available 96-well plates. Libraries are produced either as large collections of individual compounds, or as low-complexity mixtures, thus dramatically decreasing the need of deconvolution for hit structure determination while maintaining a high-synthetic throughput.

The system uses a workstation approach. A Zymate XP robotics arm distributes the plates among local robotics units performing various organic synthesis tasks, where multiple microtitre plates are processed simultaneously. The tasks include resin dispensing, reagent addition, incubation, washing and cleavage. A description of the different workstations, along with the strategies followed for libraries synthesis on solid phase and/or solution phase will be discussed.

### User-friendly top level software for controlling an automated combinatorial chemistry system

*Ton Jenneboer and Will Kuijpers, NV Organon, Oss, The Netherlands*

At the end of 1996, a fully automated robot system was installed in our research laboratories to support combinatorial chemistry activities. The heart of the system is formed by LaMOSS (Labotec Modular Organic Synthesis System), a synthesizer capable of performing liquid phase as well as solid phase chemistry. LaMOSS is based on Zymark technology. Its features include nozzle-based handling of reagents, building blocks, solvents and resin-slurry (including split/pool procedures), temperature control of the 48 reaction vessels and collection of the products in standard 16 × 100 mm tubes. These tubes can be transported with a shuttle to a Zymark XP robot where evaporation and liquid–liquid extraction can be performed.

LaMOSS is provided with pre-programmed basic functions which perform the most simple tasks (e.g. nozzle-movement, set switches). We have added so-called unit operations, each of them performing a specific task using the basic functions (e.g. add liquids, transfer tubes). Until recently, the actual synthesis was programmed in the recipe which contains a list of unit operations accompanied by the selected parameters. This recipe had to be written prior to every synthesis. The disadvantage of this set-up was the complex and time-consuming programming of the syntheses.

Initially, LaMOSS was connected as local controller to the XP-controller to enable communication between both controllers in case of tube transport. In this set-up, syntheses were programmed entirely in the LaMOSS controller using EasyLab for Windows$^{TM}$ and could be started either from there or by the XP-system. As a consequence of this set-up, manual interference in one system (stop and abort) resulted in a stop of the other system as well.

These experiences encouraged us to find a better way of controlling LaMOSS. This paper describes the development of user-friendly top level software, running on a standard PC and controlling LaMOSS through the normal EasyLab high-speed connection.

This connection became available to us with the use of Zymark Tools for Windows$^{TM}$, and relatively straightforward user-interface was developed in Visual Basic$^{TM}$ for writing and controlling a synthesis. Because this approach demands a direct connection between PC and controller, the tube-transfer to the XP-system is now organized with the use of switches and inputs in both systems.

The top level software consists of a Synthesis Editor for writing and running the recipe and an Event Viewer for showing the controller status. The chemist chooses the desired events via buttons and pop-up windows, and the program shows the recipe in 'chemical' language. Subsequently, the program adds the appropriate unit operations and parameters to be sent to the controller. In this way, the controller is handling a complete unit operation by itself, so there still is a minimum of communication time involved.

### Automated method transfer: 'a tale of two laboratories'

*Timothy A. Diehl, Janssen Pharmaceutica, Titusville, NJ, USA*

'It was the best of times, it was the worst of times', as Dickens wrote, captures the implementation process of automation systems in non-R&D laboratories. Implementing automation in a quality assurance laboratory has always been a difficult task due in part to the technical and managerial roadblocks. However, as automation becomes an integral part of many corporations' strategies to increase efficiencies, the inertia to overcome these obstacles is being gained. The use of technology transfer process helps to determine if any analytical method developed on a research instrument will work on a production instrument. However, these requirements may also complicate the implementation process. Factors both positive and negative that influence the implementation of a global automation plan will be discussed. Experiences encompassing logistics, management, regulatory, compliance, communications and technology transfer will be discussed in this presentation.

### Development and transfer of an analytical chemistry workstation to a manufacturing site

*Craig Bender, David Otto, Alan Wickman and Mark Schweitzer, Searle Research and Development, Skokie, IL, USA*

Automated analytical chemistry workstations can be very useful for a quality assurance lab at a pharmaceutical manufacturing site. Workstations can provide increased sample throughput, increased turnaround times, and more accurate and precise results. Methods can be developed by Research and Development labs and transferred to the manufacturing site on the automated equipment. Automating the analysis using the same sample preparation parameters can ensure results that agree between sites.

An automation project was undertaken by Searle Research and Development and Bohdan Automation (Mundelein, IL) to automate the analysis of dosage form assay and content uniformity testing. Two identical systems have been developed, one for Research and Development, and one for our manufacturing site in Puerto Rico. Each automated system can process up to 30 samples by serial processing using the following steps:

(1) dispense media into sample tube;
(2) vortex to dissolve;
(3) filter sample aliquot;
(4) dilute samples; and
(5) inject sample onto HPLC.

System validation and transfer activities to Puerto Rico are currently underway. Protocols and methodologies have been developed by Searle R&D. This presentation will describe the activities that have taken place during the transfer of the automated system to Puerto Rico.

### High-throughput bioanalytical mass spectrometry using 96-well solid phase extraction

*John Janiszewski, Monica Swyden and Hassan Fouda, Drug Metabolism Department, Pfizer Central Research, Groton, CT, USA*

Coupled with HPLC/atmospheric pressure ionization—mass spectrometry, microtitre 96-well plate technologies for sample and liquid handling have dramatically increased our drug metabolism bioanalytical throughput. Over the past several years, we have focused our efforts on developing and integrating several bioanalytical throughput-enhancing approaches for use in our department. These efforts have led to the development of standardized protocols for 96-well solid phase extraction methods development, assay validation and sample analysis for the quantitation of drugs and metabolites in biological fluids. The protocols feature the routine use of X-Y liquid handlers from the outset of methods development through assay validation and sample analysis. The liquid handlers are used for standard curve preparation and sample transfer to the 96-well format. Standardized dilution templates and plate layouts are used, reducing the need for customized software programming. The SPE plates are processed using a programmable 96-well pipettor, the Quadra 96. Once an analytical method has been developed, a single analyst can routinely process 400–600 samples in about 4 h. Most of the analyst's time is taken in set-up and sorting of the samples prior to and while using the workstations.

### Evaluations of validated assays using automated solid-phase extraction with LC/MS/MS for analytes in plasma

*Naijia H. Huang, Lloyd R. Whitfield, John R. Kagel and David T. Rossi, Parke-Davis Pharmaceutical Research, Division of Warner-Lambert Company, Ann Arbor, MI, USA*

Automated solid-phase extraction (SPE) using a RapidTrace workstation was developed with LC/MS/MS to qualify a drug candidate in plasma for blood lipid regulation. The validated method was characterized for precision (interday and intraday precision of 7.0% and 9.8%, respectively) and accuracy (interday and intraday relative errors of ±2.7% and ±6.7%, respectively), and recovery (greater than 90%) over a calibration range of 5–1000 ng/ml. The method was used to assay over 2500 samples, standards and controls in support of toxicology studies in mouse, rat, dog and monkey. Benefits of the automation included saving of analyst time, and decreasing exposure of analyst to biological and chemical hazards. Evaluation of automation performance (including failure rate, carryover and throughput benchmarks) will be discussed. Adaptation of the method for use with 96-well technology and insights gained during the study will be presented.

### Determination of 264W94 and 2169W94 by LC/MS/MS using a 96-well SPE: a comparison of semi-automated and fully automated approaches

*Lisa St. John-Williams, Glenn A Smith, Kathryn B. Kenney and John A. Dunn, Glaxo Wellcome, Research Triangle Park, NC, USA*

The compound 264W94 is currently under investigation as a hypercholesterolemic agent. An automated method for the quantitative determination of the drug and its demethylated metabolite, 2169W94, in human serum was developed and validated using solid phase extraction in the 96-well plate format. The extracts were analysed using electrospray ionization LC/MS/MS. The compounds are isolated from human serum using Waters' Oasis$^{TM}$ HLB extraction plates. The calibration range of the assay was 0.1–100 ng/ml.

Two different automation approaches were validated and compared. The original method was developed using a Packard Multiprobe 104DT and vacuum manifold. The method was later transferred to a fully automated Zymark XP robotics system interfaced with a Tecan RSP 8051 liquid handling workstation. The latter system uses centrifugation to carry out the solid phase extraction, and is the first example demonstrating this technique using these new robotics systems.

The validation data from both automated systems confirm that the specificity, precision and accuracy of the assay are sufficient to support the quantitation of 264W94 and 2169W94 in clinical trial samples. The problems encountered during the development of the automated methods and the validation results will be presented. The two approaches will be compared, and their unique advantages and disadvantages will be discussed.

### A comparison of pressure- and vacuum-based automated 96-well SPE systems for the LC-MS/MS bioanalysis of haloperidol in plasma

*Jaap Wieling, Jelle Hempenius, Ruud J. J. M. Steenvoorden and Jan H. G. Jonkman, Pharma Bio-Research International B. V., 9470, AE Zuidlaren, The Netherlands*

Since the introduction of liquid chromatography with tandem mass spectrometric detection (LC-MS/MS) as a quantitative technique in the field of routine bioanalysis, sample preparation became the new throughput-limiting step in the entire analytical process. Manual solid-phase extraction (SPE) is a particular example of this situation.

We have implemented various instrumentation for automated 96-well SPE (SPE96), either based on positive pressure (Hamilton Microlab) or based on negative pressure (vacuum) (Packard MultiProbe) for pipetting clinical samples, and the application of conditioning, washing and elution solvents on the solid-phase bed (3M Empore extraction plates) and subsequent flow through. To compare and validate both principles, we have implemented an SPE procedure for an LC-MS/MS method for haloperidol in human plasma on the Hamilton and Packard systems, keeping the experimental conditions for the extraction on the two systems as similar as possible. Besides validation results on precision and accuracy, some other performance aspects were assessed, e.g. throughput, number of assays to be repeated due to blockings, human intervention, detection of levels and carry-over.

The validated calibration range was from 0.100 to 50.0 ng/ml. The inaccuracy and imprecision were below 15% at all concentration levels and for both systems, although the coefficients of variation for the vacuum system were significantly better (two-sided $F$-test). This was most likely caused by the Hamilton's flow detection system using a pressure-drop monitor, leading to drying out of the wells and subsequent channelling and irregular wetting of disks, and thus less reproducibility within an SPE96 plate. Secondly, the larger carry-over of the pipetting equipment of the Hamilton system caused less precision.

Although the sample throughput of the Packard systems may be higher as a result of a four-needle configuration, current commercial systems cannot operate fully unattended; technicians must interfere by changing 96-well plates, positioning sample collectors underneath the plates, and transfer of the sample collectors to the HPLC systems. The Hamilton operates slower (current system applies only one needle), but operates fully unattended.

### Allegro™: moving the bar upwards

*Mary Jo Wildey, R. W. Johnson Pharmaceutical Research Inst., Raritan, NJ, USA, Carol Ann Homon, Boehringer Ingelheim Pharmaceuticals, Ridgefield, CT, USA, and Burleigh Hutchins, Zymark Corp., Hopkinton, MA, USA*

At the 1995 Society for Biomolecular Screening Conference, the term ultra-high-throughput screening (UHTS) was introduced. UHTS was to be the next generation of screening and was defined as the screening of 100 000 samples in an assay in a day. This new rate of screening would be required to test the million-compound libraries enhanced through combinatorial chemistry of the future. At that time, most highly automated systems had a throughput of 100 plates a day. The major limitations for these systems were the robotic designs and the time of analysis. Cost was to be a major factor for the screening of these new million-compound libraries. To maintain costs, new technologies would need to be incorporated into the screening solution. Miniaturization and nanotechnology became the new buzzwords. However, it soon became evident that even conversion from 96-well formats to 384 was not going to be as simple as expected. New designs for 384-well microtitre plates are evolving to address the problems identified with the earlier designs, e.g. wicking and pipetting with multiple tips. It was appearing that UHTS would have to wait for better plate designs, remodelled pipettors to specifically address 384-well formats and lower pipetting volumes, and changes in quantitation techniques to imaging. In the meantime, the issue of how to manage and move the plates required for 100 000 compounds needed to be addressed. In the fall of 1996, an idea emerged around the simple concept of platemovers™ instead of robots, and reducing the HTS process to a series of individual steps separated by time. A robotic concept now called Allegro™ by Zymark would be a series of fully independent modules working together in a process-like manner very similar to an assembly line. The simple motions required of the platemovers™ would allow them to move faster, make them far more dependable and much easier to program. These modules could be easily reordered, passed or linked to create new procedures. The system would be fully enclosed to allow control of the assay environment in terms of gas, temperature and humidity. This new modular robotic system completely processes 1200 plates of a 96-well design in 24 h. Allegro™ will also allow for easy incorporation of new technology-based modules. As this new technology is incorporated, the future throughput in terms of total number of wells could be even higher. Once Allegro™ has finished its initial test programs, assays that are compatible with a 384 format will be started. This presentation will present the Allegro™ system applications as well as an evaluation of this new concept as it pertains to performing UHTS and its integration in the research environment.

### Cell-based assay miniaturization for ultra-high-throughput screening

*Ilona Kariv, Anthony M. Maffia and Kevin R. Oldenburg, DuPont Merck Pharmaceuticals, Wilmington, DE, USA*

Recent advancements in the fields of combinatorial chemistry and genomics greatly increased the size of chemical libraries and the number of targets against which they are screened. This substantial growth in library size and target number necessitates changes in high-throughput (HTS) screening. One solution is to miniaturize the HTS format, thus providing a possibility to increase throughput and save reagent cost. Miniaturization involves designing and adapting current HTS assays to high-density formats, either 1536-well (5 μl) plate for cell-based assays or 6144-well (0.7 μl) plate for soluble enzyme assays. Although some progress has been achieved in the last few years in adapting bacterial and protein-based screens to high-density formats, mammalian cell-based assay modification still remains a major challenge. One of the primary obstacles is the ability to dispense cells in a small volume, while maintaining cell integrity. This is especially true for functional assays relying on transcriptional machinery to express genes of interest. Extensive assay run time creates the additional problem of liquid evaporation. In this study, we selected a cell-based assay utilizing luciferase reporter gene under control of the gene of interest 5′ binding site in a stably transfected Jurkat T cell line, as a model. The objective was to identify specific inhibitors of the expression of the gene of interest. Limitations and advantages in adapting this assay to high-density format, 1536-well plate, and technical considerations and challenges are further discussed in this report.

### ExtremeScreen™: meeting the challenges of ultra-high-throughput screening

*Michelle Palmer, Mark Roskey, Bonnie Tillotson, Neil Carlson, Mike Nemzek and Irena Bronstein, PE Tropix, Bedford, MA, USA*

Extreme high-throughput screening (EHTS) at rates beyond 100 000 compounds/day is an emerging technology. It is difficult for a screening laboratory to dedicate resources to all the new screening technologies and incorporate them into their working environment. The challenges of EHTS include selection of technologies and formats, the associated learning curve, adaptation of existing assay methodologies to new formats, and required infrastructure and logistic issues.

Tropix ExtremeScreen™ is a new concept that provides a three-tiered approach to delivery advanced chemiluminescent screening technologies. First, ExtremeScreen provides proprietary chemiluminescent dioxetane screening reagents to meet the stringent demands of yielding high-quality data across a wide range of assay types in a single platform at very high-throughput levels in a cost-effective manner. Second, ExtremeScreen rapidly develops custom screening assays for deployment on screening systems at the user's site. Third, ExtremeScreen is a complete service to perform advanced screens at rates above 100 000 samples per day with in-house or customer-supplied libraries, bringing extremely rapid turnaround on large or small screening projects. The combination of the three elements significantly contributes to accelerating the drug discovery timeline.

### Technology, logistics and common sense: challenges and opportunities in high-throughput screening

*John Major, Lead Discovery Department, Zeneca Pharmaceuticals, Alderley Park, Macclesfield, Cheshire, UK*

High-throughput screening (HTS) is a key process in pharmaceutical lead identification. Screening throughputs of tens of thousands of compounds per assay run are typical. Advances in chemistry are providing opportunities to access increasingly large compound sets. This development is fuelling a trend towards greatly increased screening capability. Effective systems need to be devised for storage, retrieval, formulation and supply of compounds to screens. HTS operations need to be fuelled with novel drug targets, and screening systems capable of efficiently delivering leads rather than hits are urgently required. There is a need to deploy the principles of high-throughput screening against downstream functional biology and the drug adsorption, metabolism and toxicology bottlenecks. These requirements present significant challenges in relation to assay technology, screening logistics, automation and management of the processes. The challenges and the opportunities they present will be discussed.

### Leveraging a discovery organization: the Sepracor experience

*James R. Hauske, Sepracor, Marlborough, MA, USA*

Discovery at Sepracor is shaped by the strategic imperatives of the ICE® portfolio, i.e. our drug discovery efforts are directed toward the therapy areas of inflammation, pain and urological diseases. We have attempted to enrich the highly successful ICE® approach by broadly defining each of the therapeutic areas to include a wide range of molecular targets with potential therapeutic advantage over existing approaches. These approaches are ultimately directed at disease with unmet, undermet, medical need and significantly large patient populations. By focusing our efforts on the core competencies of synthetic chemistry, medicinal chemistry and combinatorial chemistry, we have developed a proprietary and highly diverse corporate file of small, drug-like molecules. Our strategy of developing collaborative relationship with selected biotech partners with proprietary molecular targets, or proprietary platform technology, in the therapeutic areas of strategic interest to Sepracor provides a means to most effectively leverage our ever-expanding corporate file of drug-like molecules. Combining our unique skills in combinatorial chemistry and medicinal chemistry with the highly evolved HTS competency and proprietary molecular targets of each of our biology-rich collaborators has driven the rapid implementation of our drug discovery strategy. We will describe the approach that we utilized to define the portfolio of Sepracor Discovery, as well as the implementation of the discovery programs.

### Leveraging your technology investment: maintaining the competitive edge in drug discovery

*Ralph Infantino, Janine Schwedes, Margaret Reynolds, Mimi Rivera and Mel Reichman, OSI Pharmaceuticals, Uniondale, NY, USA*

Recent advances in drug discovery technologies present a wider range of opportunities that require careful evaluation for compatibility with existing systems. This can be an arduous task for an established facility with much invested in the current infrastructure. Because HTS is no longer the rate-limiting factor in discovery, established R&D sites specializing in it must carefully weigh its options and aim for rapid return on investment. Issues that must factor into decisions on increasing throughput and capacity must address questions involving how quickly a screen must be completed versus the pace at which assays are formatted and validated. Moreover, solutions should address how to leverage the resources required for lead follow-up, medicinal chemistry, compound acquisition and pharmacology. We will describe our philosophy and operational approaches that allow us to derive maximum value from our formidable existing infrastructure dedicated to HTS, while keeping pace with emerging technologies ad the ever-increasing need to increase throughput and capacity.

### An overview of the methods validation process

*Patrick J. Faustino, Center for Drug Evaluation and Research, Office of Pharmaceutical Science, Division of Product Quality Research, Laurel, MD, USA*

The methods validation process involves three areas: validation, evaluation and verification. These terms are defined by the Analytical Methodology Technical Committee (AMTC) of the FDA within the current revision under 'good guidance practices' (GGPs) of the 1987 guidance: 'Guideline for submitting samples and analytical data for methods validation'. The three areas outline the regulatory responsibility of the drug sponsor and the agency. Validation is the responsibility of the drug sponsor to prove the analytical method will work according to its intended purpose. Evaluation is the responsibility of the review chemist. Evaluation is the analysis of the drug sponsor's validation of the analytical method. Evaluation also defines what analytical procedures and performance parameters are required and specified in the 1987 guidance and the USP chapter 1225. Verification is the responsibility of two FDA field laboratories to establish the suitability of the method for regulatory purposes. Further discussion of the methods validation process will also include ICH guidelines and FDA's Chemistry Manufacturing and Controls committee's review practice and guidance development.

### Development and validation guidelines for automated methods automation subgroup of the pharmaceutical and analytical sciences group

*John Stanley, SmithKline Beecham Pharmaceuticals, Harlow, Essex, UK*

The Automation subgroup of the PASG in the UK was set up at the beginning of 1996 and has since grown to include representatives from pharmaceutical companies, vendors and contract laboratories. The purpose of the group was to exchange experiences on robotics and to influence vendor developments. However, it soon became clear that the most immediate need, and probably the greatest challenge, was to construct guidelines for the development and validation of automated methods. Individual companies had gone through parallel development processes that relied on hard won experiences and considerable expertise. The novice robot user is faced with a very steep learning curve, so considerable effort could be saved by the pooling together of the collective expertise already available in the group.

The resulting document contains detailed recommendations for single and composite dosage form assays, degradation/impurity profiles and dissolution testing, and is suitable for any instrument that is capable of performing these tests in a laboratory environment. The presentation highlights the important issues arising from the work of the PASG automation subgroup.

### Automated method validation of an oral product's assay and content uniformity

*Samuel Barrera, Rafael Cruz, David León and Carlos Rivera, Bristol-Myers Squibb Company Bristol Laboratories Corporation, Quality Control, Mayaguez, PR, USA*

QC challenges for improving productivity, reducing cycle time, improving efficiency and maintaining a high level of regulatory compliance require a high application of advanced technology. On the other hand, the concentrated efforts of AR&D in drug discovery, a fast pace of newly launched products and the constraints of cost reduction have led to more independence of QC labs in method validations. This situation triggered the QC initiative undertaken at Bristol Laboratories Corporation in Mayaguez, PR to validate the TPWI workstation for the automated sample preparation of BuSpar assay and content uniformity based on previous applications of the QC lab in Evansville of this same company. This project has opened the door for other applications that will result in higher throughput, efficiency and other benefits of technology for success.

### Keys to successful method validation and method transfer in the R&D environment

*Jon P. Sadowitz and Sam Li, Barr Laboratories, Pomona, NY, USA*

Automated techniques for sample testing are readily increasing in the R&D environment. With this increase comes the need for pertinent validation that streamlines the path for use of these techniques, and addresses the means to transfer these methods to other areas or departments, either locally or remotely. This presentation will address the prevailing philosophies, techniques and protocols of the R&D automated method development team, and how they affect the automated method validation of solid dosage forms of assay, content uniformity and impurities testing along with the transfer of these methods to the QC environment.

### Automation and robotics—the key elements in global standardization of physical test methods

*Ramasamy Tamilarasan, Paul L. Morabito and Jonathan J. Zieman, The Dow Chemical Company, Midland, MI, USA; Paul D. Hazelwood, The Dow Chemical Company, Sarnia, Ontario, Canada*

Companies have realized that they have to embrace the world as their workplace and marketplace in order to grow and compete in the global market economy. They are sprinting into the global marketplace at record clip, propelled by technology. The next-generation companies will be so integrated with the world that they will be non-national rather than multinational.

Globalization of products is forcing the companies to globally standardize the production and quality control test methods. This paper describes the development and implementation of the robotic automated systems for tensile, FLEXURAL, hottack and heat seal tests of polymeric materials. These systems handle rigid polymer samples of 6–8 inches long, polymer film samples of 6–13

inches long, as thin as 0.001 inch. The other capabilities of the systems include reading barcode labels, measuring sample thickness and communicating to the testing instrument. These automated systems enabled the global standardization of the test methods, improve the productivity by nearly 150%, and provide consistent and quality results in a cost-effective manner.

### A new standard for polyurethane physical property testing

*Steven E. Robbins and Michael E. Rusak, Air Products and Chemicals, Allentown, PA, USA*

Polyurethane foam is found in hundreds of applications, including furniture cushions, automotive seating, appliance and construction insulation, and carpet underlay. Polyurethane foams are created by reaching isocyanate, water and polyol in the presence of catalysts, surfactants and potentially other additives. The resultant polymer morphology resembles a sponge, a non-homogeneous material with a varying cell size distribution. While the cellular nature of the foams is exploited to meet certain performance characteristics, physical property measurements must be based on samples large enough to represent average behaviour.

Industry standards, e.g. ASTM, define sample handling, conditioning, shape and size, as well as manual testing methods, for each physical property desired. Our experience has shown that these manual methods are subject to operator and device variability.

During the past decade, we have developed standard automation approaches to improve sample handling, instrument control and data acquisition, while developing a collection of reusable hardware and software components. Data reliability and reproducibility have improved greatly for each of the methods we have automated. These methods include density, airflow, compression set, Japanese wet set, tensile-tear, and dimensional stability. This presentation will illustrate test improvements and some of the automated techniques developed to achieve them.

### Custom bar code label printing software for laboratory automation

*Saul Llamas and Stephen Dokoupil, Automation Laboratory, Helene Curtis Innovation Center, Unilever Home and Personal Care, Rolling Meadows, IL, USA*

Barcodes are prominently used in the retail marketplace and have found many uses in other fields, including the area of laboratory automation. Jars, prototype containers, test tubes, sample vials and microtitre plates are just a few of the items that are now bar coded. Bar codes are key to a successful robotics automation system. Bar codes allow a robotics system to identify and track samples. Bar codes also enable the use of embedded protocols within the bar code number that identify the tests to perform on a sample. In addition, bar code labels allow placement of human-readable data, providing users in the laboratory with quick descriptive information on a particular test sample.

Two approaches used by Helene Curtis for printing bar code labels will be presented. The software presented are two custom bar code label printing applications developed in-house; one written in LabVIEW™ and the other in Visual Basic™. A comparison of LabVIEW™ and Visual Basic™ software for application development will be discussed, along with techniques on interfacing custom software to the Zebra™ bar code printer. Interfacing databases with the bar code software will also be covered.

### An automated sample preparation for determination of total siloxanes in shampoo products using inductively coupled plasma (ICP) spectroscopy

*Kyoko Ida and M. Karita, Procter & Gamble Far East, Higashinada-ku, Kobe, Japan*

This paper describes an automated sample preparation for the determination of total siloxanes in shampoo products using inductively coupled plasma (ICP) spectroscopy. A fully automated robotics system requires only setting blank sample tubes on the racks and putting products into these tubes manually. Robot preparation gives us higher efficiency in extraction and lower RSD than current manual preparation.

Currently, we can analyse 30 samples per day by the manual method at KTC. However, our new automated robotics method is expected to have the capability of analysing 50 samples per day. Therefore, this automated robotics method will supply us with much higher speed for determination of the siloxane percentage in shampoo products.

The analytical data sets demonstrate accuracy, precision and reproducibility of the sample preparation. Linearity, accuracy and precision ensure the validity of the robotics method. The method has a linearity working range from 0 to 500 ppm (correlation coefficient, $R = 0.9999$). The method gives sufficient recoveries, $R = 101 \pm 1\%$. Relative standard deviation (RSD) is 0.42%.

### Database mining and knowledge discovery in modern drug research

*G. M. Maggiora, M. S. Lajiness, D. W. Elrod, M. A. Johnson and T. R. Hagadone, Pharmacia & Upjohn, 301 Henrietta Street, Kalamazoo, MI, USA*

A growing trend towards automation within the pharmaceutical industry has led to an explosive growth in the rate, amount and types of data that must be dealt with in modern drug research. Within the space of only a few years, highly sophisticated technologies have transformed drug screening; DNA, RNA and protein sequencing; and chemical synthesis increasing throughput in these key areas by a thousand-fold or more. Computers provide the only effective means for managing this growing flood of data. But managing data is not enough if the knowledge embedded within it is to be obtained. It is here that newly emerging techniques of database mining and knowledge discovery, already having considerable impact in many areas of business and industry, may also play an important role in pharmaceutical research. In high-throughput screening, e.g. numerous biological assays are run against corporate compound collections typically of several hundred-thousand compounds or more. Such a database represents a watershed of undiscovered biological knowledge, but the database must be properly 'mined' if the knowledge within is to be realized. The discovery of relationships, rules and patterns in databases of this size is, however, a daunting task at best and cannot be addressed with many of the tools used successfully on much smaller datasets. The talk will present a brief overview of the salient issues in database mining and knowledge discovery that are applicable to pharmaceutical research. Several examples will be provided that illustrate how these methodologies can be used to extract new knowledge that can potentially expedite the drug discovery process.

### Quality control for high-throughput screening

*Michael W. Lutz and Jay Gill, Glaxo Wellcome, Research Triangle Park, NC, USA*

Robotic systems for biochemical assay introduce a new set of requirements for analysis of quality control data. The precision and accuracy of liquid dispensing robots and complete robotic assay systems must be determined and monitored. To properly interpret data, assay variability must be quantified. Automatic detection of gross errors, systematic errors and random errors is necessary for systems that run continuously.

This presentation addresses statistical methods and practical concerns involved in quality control analysis for high-throughput screening. After a review of methods, examples are given that demonstrate how quality control statistics are applied in the area of high-throughput screening. Finally, the recommendations for best practices are discussed.

### Maximizing information content in combinatorial libraries

*Steven L. Gallion, ArQule, Medford, MA, USA*

The process of library design and interpretation of SAR data should be guided by the ability to extract substantive information during each design cycle. The successful management of data during the entire drug discovery process is a function of the ability to not only track and store data, but to formulate and refine hypotheses that result in at least a semi-quantitative rationale for lead optimization. This presentation will discuss the issues and strategies involved in the design of combinatorial libraries to deliver maximal information content. Examples include the rational selection of building block components using a combination of medicinal chemistry expertise, computational analyses, crystallographic structures and diversity assessment tools.

### From functional genomics to prototype drugs

*Dale S. Dhanoa, 3-Dimensional Pharmaceuticals, Exton, PA, USA*

Advances in genomics continue to discover new exploratory targets for drug development in large numbers. At 3-Dimensional Pharmaceuticals, we have developed a powerful drug discovery system that integrates our proprietary technology components of Probe Libraries, ThermoFluor® = AE High-Throughput Screening, Directed Diversity® = AE and Informatics. The strategy for finding small molecule prototype drugs for functional denomics targets using our integrated drug discovery systems will be described.

### Inhalation product testing: automated sample recovery from Cascade Impactors

*Phil Waters, Glaxo Wellcome, Research Triangle Park, NC, USA*

The Andersen eight-stage Cascade Impactor is a standard device used to characterize the aerodynamic particle size distribution of metered dose inhaler (MDI) and multiple dose powder inhaler (MDPI) products. The manual test typically requires assembly, dosing and separation of the Impactor unit; rinsing each stage/plate component into separate collection containers; adding sufficient volume of solvent; and sample assay (typically by HPLC). This paper discusses a device developed by Glaxo Wellcome that automates the time-consuming rinse steps associated with the recovery of drug from Cascade Impactor stages. A detailed description of the hardware and software configuration is presented, along with analytical validation results for products currently tested using this system.

### Enhanced decision-making from analytical automation

*Harnath Doddapaneni and John Jushchyshyn, SmithKline Beecham Pharmaceuticals Research & Development, Upper Merion, PA, USA*

Automation initiatives have been traditionally viewed as a resource-reducing tool. The ability of automation to generate high-quality, reproducible and correlative data has been largely overlooked. This paper will present long-term dissolution data generated on development batches of drug product which compare manual approaches versus a segmented automated approach. The data produced using automated methods reduce or eliminate variation due to slight changes in manual techniques which occur from analyst to analyst over a period of years in a typical stability study. While the outcome of a stability exercise is equivalent, the data produced using automated procedures allow management decision-making at a much earlier stage in the stability cycle when compared to other approaches which are inherently more variable.

### Development of an automated assay for sustained release tablets using the TPW-II Workstation

*John C. Lynch, Zeneca Pharmaceuticals, 1800 Concord Pike, Wilmington, DE, USA*

Automated composite and content uniformity assays for sustained release tablets have been developed using the

TPW-II Workstation. Use of the TPW-II has reduced sample prepartation and analysis time from over 4 h per sample to under 30 min per sample, significantly increasing sample throughput. Because the TPW-II homogenizes, filter and dilutes each sample, analysts spend less time preparing samples and can focus on other responsibilities.

In order to successfully and efficiently automate methods using the TPW-II, analysts need to evaluate the capabilities and limitations of the workstation as well as the method development costs versus the advantages (throughput, labour, safety) achieved by automating a specific assay.

Our experience developing automated sustained release assays has enabled us to develop a method development template for the TPW-II. A simple, step-wise approach has been defined to guide automated method development and provide the analyst with a reasonable estimate of the work required at any given stage of product development. This presentation will discuss this method development approach using specific examples from the automated sustained release tablet assay.

### Development of an automated liquid store to improve sample management in the lead discovery process

*Sue Holland, Peter Aperghis, Melanie O'Neill, Clive Battle, Tim Linsdell and Martin Sykes, Glaxo Wellcome Research & Development, Medicines Research Centre, Stevenage, Herts, UK*

The potential for identifying new lead compounds in the drug discovery process is increasing through the use of faster and more efficient approaches to high-throughput screening (HTS) and larger, more diverse compound collections. The rapid developments in robotic HTS systems and the expansion of sample libraries through combinatorial chemistry place demands on traditional approaches to sample supply which have made this element of the lead identification process rate limiting.

In order to maintain pace with HTS, a sample supply strategy is required which will respond rapidly to screening requests, provide the flexibility screeners want, and maintain control and accountability for the quality of samples produced. To achieve these goals, Glaxo Wellcome has developed an automated sample storage, retrieval and processing system with the capacity of 3 million samples and an output rate of 15 million samples per year. The automated liquid store (ALS) comprises an environmentally conditioned sample storage facility and an input/output buffer integrated with three liquid handling robots (LHRs). The system will perform sample replication and discrete sample picking from 384-well source blocks into either 96- or 384-well microtitre plates.

The scale of this facility has called for an industrial design concept which is modular to allow reconfiguration of the LHRs to meet future business needs whilst maintaining the core storage and retrieval infrastructure. This presentation will review the role of the ALS in the GW sample supply strategy, its principle elements and functions, and the approach taken to managing the project.

### A robotic compound dissolution system for the Pearl River Research Compound Bank

*Kevin Olsen, Robotocist, Research Compound Bank, Wyeth Ayerst Research, Pearl River, NY, USA*

In order to make more compounds available to high-throughput screening researchers, the Pearl River Research Compound Bank and the Wyeth Ayerst Biomedical Engineering Department collaborated on the construction of compound dissolution and distribution robots.

The machines are modified industrial gantry robots and are controlled with a Visual Basic interface running on a PC. Other than the basic robot, all hardware and software development was developed by Biomedical Engineering. By standardizing upon and then modifying a basic platform, the engineers used this project to develop the expertise that was applied to subsequent projects.

The machines were designed to process 92 compounds per hour. Data entry is completely automated through the use of bar codes on both the compound samples and microplates. Liquid handling was engineered so that dissolved compounds would be completely utilized, and there would be no residual aliquots at the end of the run. The units have been extremely reliable. During the fall of 1996, they compiled a record of only three mechanical failures in 75 000 samples.

### Validation of 1 μl compound delivery to dry plates using the Zymark RapidPlate

*Brent T. Butler, Joan Frezza and Julie J. Tomlinson, Department of Molecular Biochemistry, Glaxo Wellcome, Research Triangle Park, NC, USA*

The quality of data generated in screens may be compromised by the instability of compounds delivered to those screens. 'Just in time' dilution of compounds will help to maintain the stability of these compounds. The ability to delivery 1 μl of compound in neat DMSO to a variety of target plates is the basis for just in time compound delivery and paramount to increasing the quality of screening data generated in those targets. This also eliminates a dilution step in the compound handling system, thus decreasing the time spent preparing compound plates for the numerous targets being screened.

Results of experiments on various plate types indicate that Zymark RapidPlates located on the compound handling robot in the Molecular Biochemistry Department, RTP are able to dispense 1 μl of DMSO reliably and accurately. The c.v. for all plates tested were less then 10%, with accuracy between 95 and 110%. This capability will allow compounds to be delivered to targets just in time, which will help to maintain the stability of these compounds. The compound handling robot will run control plates at regular intervals to monitor RapidPlate function.

### Use of a Zymark XP system for multiple compound formatting tasks

*Richard Spann, Molecular Pharmacology, Berlex Bioscience Richmond, CA, USA*

The Zymark XP laboratory automation system provides a versatile technology that can be fully upgraded with the latest enhancements at desired intervals. The modules include a Zymark XP arm, a Master Lab Station II, a Power and Event Controller, a Fully Automated Capping Station, a RapidPlate-96 and two Storage Carousels.

The architecture is standard System V with operating system version 2.5. The interface PC is running Windows and the Zymark Utilities package. Previous versions of the Zymark OS only allowed I/O interactions with the System V controller disk drive or selected files on the hard drive via Easylink. Using functions supported under the Zymark Utilities, data I/O is now possible directly with any available drive.

The system is being used to support compound formatting and distribution for high-throughput screening (HTS). The setup can perform four different activities: dissolution of vials of compound then transfer to 96-well plates; transfer from vials of solubilized compounds to 96-well plates; dissolution of compounds in vials; and consolidation of plates containing single compounds per well into plates containing 10 compounds per well. The plates with 10 compounds/well can then be used in HTS assays. The matrix strategy employed allows for an efficient means to identify HTS hits.

### Laboratory data management in the QA environment

*Tricia Chen, Novartis Pharmaceuticals, E. Hanover, NJ, USA*

Data management is a significant challenge for the Pharmaceutical Quality Assurance organization. The myriad of regulations imposed by the FDA and DEA and their subsequent translation into business practice affords the industry many technical challenges with respect to managing the data. From the point of data generation in its completely raw form to its fully processed and final resting places, the data undergo several transformations and multiple moves.

At the lowest level, the commonplace business response is to utilize a data acquisition system to control the analytical instruments and to ensure that the collection of laboratory data follows strictly defined (in-house) guidelines. The twin concerns of data integrity and auditing can then be addressed by relegating a collection of routine low-level tasks (e.g. data entry, checking, storage and retrieval) to the discipline imposed by computer processes. For better control and efficient maintenance, a networked data acquisition system is recommended strongly instead of stand-alone integrators.

At the next level, we find the LIMS software with its two primary roles, i.e. to coordinate/orchestrate the testing and to centralize the generated data. When all three stages of the process are properly structured, i.e. (i) the automation of data captures; (ii) the transfer of the data via networked (interfaced) laboratory instruments; and (iii) the centralized storage of the test data, the auditability and trace-ability issues surrounding the data capture can be fully addressed. This approach to data integrity can provide management with a high degree of confidence in their business data, and the ability to respond in a timely manner to external regulatory agencies.

At the third (top) level, the global needs of the business can be addressed. Today's business climate is fraught with corporate mergers, increasing process regulation, new reporting constraints, and software that simply does not cut it anymore. All of these circumstances are compelling from the standpoint of initiating business process re-engineering projects. Projects directed at the data collection level preserve the viability of the business on the day-to-day operational level and serve to lay the groundwork for utilizing the data in more creative ways. Projected directed at cross-department sharing of data (e.g. QA laboratory data with that of manufacturing), or data sharing globally within a department serve to create new business efficiencies. Such large-scale projects, e.g. those of data warehousing, can be viewed as both strategic and defensive when consideration is given to the preservation of historical data.

### Utilizing ACCESS to efficiently manage data in a quality control microbiology laboratory

*C. R. Bierman and T. M. Kajs, The Procter & Gamble Company, Mason, OH, USA*

The quality control constituent of the Health Care Microbiology Department at Procter & Gamble is responsible for microbial content testing (MCT) and preservative challenge testing (MST) of all health care products developed at the facility. MCT and MST are required testing for product release and expiration data determination. Put simply, data accumulated from these two analyses, over time, determine the microbial stability profile for each health care product. These data are stored in the data documentation.

DDR is a system built on ACCESS software, which manages MST and MCT data in an electronic, fully searchable format. It was developed using the collaborative efforts of the Microbiology Department and Computer Management Systems.

Prior to the inception of DDR, the department tracked all data in hard copy. As tests were completed and verified, data for each sample were entered into a control laboratory information management system. Data entry proved to be time consuming and cumbersome. This system did not offer a means of searching MST and MCT data to the microbiologists, the primary owners of the information. Microbiology's need for capturing total products stability was critical; therefore, the DDR system was developed to help streamline work processes, increase the department's data management capability, and provide electronic retrieval capability.

### Using a custom-designed compound tracking system to facilitate the automation of the production of the Optiverse™ lead generation library

*L. Cameron, C. Garr and L. Schultz, MDS-Panlabs, 11804 North Creek Parkway South, Bothwell, WA, USA*

The desire for faster drug discovery led to the implementation of combinatorial methodologies for small-molecule synthesis. In 1995, MDS-Panlabs and Tripos began a collaboration to produce the Optiverse$^{TM}$ Library, a parallel synthesis library composed of > 140 000 diversity designed compounds. We have implemented a purification process such that highly purified compounds will join the Optiverse$^{TM}$ suite of compounds. In doing so, the information explosion associated with the process requires special attention. The challenge becomes tracking this information and maintaining the compound–data link which is accomplished using a custom-designed compound tracking system (CTS) database. The focus of this presentation will be on the CTS and how it facilitates the modular high-throughput processes.

### Integrating good statistical practice (GSP) in HTS information management

*Frances P. Stewart, SmithKline Beecham Pharmaceuticals R&D, King of Prussia, PA, USA*

The integrity of assay data is a quintessential construct in the success of the HTS paradigm in drug discovery. As technology increases in both high-throughput combinatorial synthesis and ultra-high-throughput screening, assay information management must evolve to address the increasing amounts of assay data. The software integrated with laboratory robotics to capture and store the data must include the functionality to alert the user when statistical tests detect erratic results or shifts due to poor process control or instability of reagents across time. At SmithKline Beecham Pharm., the IT infrastructure that supports high-throughput screening implements components within our screening data management application that monitor the quality of the data and alerts users to intervene when poor data quality is suspected. Baseline control limits are established for all HTS assays as part of the development criteria before these assays progress to the 'live' status of production runs. These limits are used to flag bad data, which enables users to cancel the data and continue screening or to suspend screening until the source of the problems are identified and corrected. This ensures that false positive/negative rates are minimized and that data posted to the corporate DB is valid. Our philosophy towards data regulation and an overview of our screening data management software are discussed.

### The development of a dynamic user interface for drug discovery operations

*Ralph Infantino, Ildefonso Belliard, Chris Waller and Mel Reichman, OSI Pharmaceuticals, Uniondale, NY, USA*

Data management for drug discovery poses a variety of challenges to an informatics group. Unlike certain 'mature' classic business operations, e.g. accounting or purchasing, the operations model for drug discovery is in its infancy and varies greatly from one company to the next. Commercial packages for HTS data management require substantial customization. As needs change, a continuous cycle of applications development follows. The information management system that complements drug discovery operations must keep pace with the dynamic nature of R&D. We will provide an overview of our drug discovery informatics system. The modules are developed utilizing a combination of industry-standard development tools, third-party components, Oracle and ISIS as a database back-end, and a novel interface concept. This system is meeting our current needs to track HTS and associated downstream data flow. It is also poised to meet future requirements for closer tracking of lead progression.

### The use of TRAP for lead structure identification

*M. Kozlowski, Telik, S. San Francisco, CA, USA*

TRAP is a proprietary technology that is based on understanding the biochemical description of a molecule and using that information in concert with structural descriptors to minimize the number of assays required to find lead compounds for pharmaceutical targets. TRAP complements the drug discovery process by providing an efficient and inexpensive way to identify lead compounds for targets that are not amenable to a high-throughput screening (HTS) format. TRAP is also an efficient way to identify lead compounds and validate targets that have little pharmacological data. Terrapin employs TRAP, as well as computational, medicinal and combinatorial chemistry, to find families of high-affinity compounds for pharmaceutical targets. The TRAP biochemical description of a molecule is derived from experimental data. Competitive binding fluorescence polarization (FP) assays are used to determine the affinities of compound for a diverse panel of proteins. A high-throughput screening system has been designed around our FP assays to ensure the rapid addition of affinity data to our TRAP database. This presentation describes the unique aspects of Terrapin's process of lead structure identification.

### Discovery and optimization with large encoded combinatorial libraries

*Daniel Chelsky, Pharmacopeia, Cranbury, NJ, USA*

Pharmacopeia has produced over four million compounds using its ECLIPS encoding technology. These compounds have been synthesized as approximately 85 discrete sets of 'libraries' which can provide substantial SAR information at both the discovery and optimization phases of a program. Information obtained is not limited to the primary target and can be extended to selectivity as well as preliminary toxicity and metabolism, allowing early choices among related compounds in a series. Case studies as well as the integration of 1536-microwell technologies will be described.

### Making the move to 384-well high-throughput screening

*J. LaRocque, B. Czermik, N. Orlova and J. Babiak, Wyeth-Ayerst Research, Pearl River, NY, USA*

Recent advances in automation and assay detection make the use of 384-well plates a viable solution to increase efficiency in high-throughput screening. We have evaluated the use of 384-well plates to run a cell-based receptor antagonist assay and an *in vitro* enzyme inhibition assay. A comparison of each assay run in 96-well and 384-well plates with regard to assay performance, automation, reagent usage, efficiency and throughput will be presented.

### A second-generation approach to molecular diversity

*J. Christopher Phelan, Axys, South San Francisco, CA, USA*

The increasing sophistication and diversity of chemical structures available through combinatorial chemistry commensurately increases the challenge of how to choose which compounds to make given limited resources. We will discuss the problem of molecular diversity in large screening libraries for lead discovery. We will present a new 'hybrid' approach for simultaneous optimization of molecular dissimilarity (a 'negative' property) and describe drug-like parameters ('positive' properties)

### Universal solvent exchanging device and reaction optimization tool in organic synthesis

*Ilya Feygin and Leslie Walling, Pharmacopeia, Monmouth Junction, NJ, USA*

Reliable and fully programmable delivery and evacuation of solvents and reagents into and out of the reaction vessels remain to be two of the biggest challenges in automated chemical research. Another major challenge is providing controllable temperature and level of agitation independently in each of the vessels.

Results of a joint effort between chemists and engineers at Pharmacopeia that successfully resolved these issues will be discussed.

A novel device with an open frame architecture, and individually observable and accessible vessels allows for either separate or batch processing of reactions. The device provides controlled temperature, programmable selection of delivered solvents and reagents, and three different modes of agitation.

A novel side-stirring mechanism placed on individual vessels creates a controllable, two-dimensional vortex capable of effectively agitating different resins.

The rotating vessel-holding carousel provides a reliable circular alignment and engagement with the stationary top and bottom manifolds. This new technique of filling and draining vessels which was enhanced by special mating fittings has allowed for elimination of liquid handling valves, reduction of tubing and the increase in overall reliability of the system. This device is currently going through laboratory testing, and it will most likely be commercialized later this year.

### High-throughput automated organic synthesis using the myriad core system

*Nick Hird and Bill MacLachlan, SmithKline Beecham Pharmaceuticals, Harlow, Essex, UK*

The myriad core system (MCS) is a new automated synthesizer developed by a consortia of pharmaceutical companies and The Technology Partnership (TTP). It is based on a flexible modular design and has capability for both high-performance and high-throughput chemical synthesis. The system incorporates several novel technologies in reaction vessel design, reagent delivery and inert gas blanketing and is controlled by user-friendly software that enables multi-job scheduling. A description of the MCS and its use in the synthesis of solid and solution phase libraries will be presented.

### A chemist's simple approach to robot programming for reaction optimization: scheduling non-identical procedures

*Robert Osborne, Zeneca Pharmaceuticals, Hallen, Bristol, UK*

The use of statistical experimental design in the optimization of chemical processes requires the performance of many pre-planned repetitive experiments and hence is an obvious candidate for automation. The programming of such automated sets of experiments is complicated by the need to vary conditions (temperature, time, addition rate, etc.) between individual experiments as well as variation in the reagents used. This paper will present a view of the particular requirements for the automation of process development compared to lead optimization and will present one simple approach, using standard software, to providing the flexibility required for successful automation in this area.

### Automated dose measurement stations—valuable players in the development of Turbuhaler® inhalers

*Roger Appelqvist, Peter Andersson, Hans Lundbäck, Thomas Lööf, and Bo Olsson, Astra Draco AB, Lund, Sweden*

Turbuhaler is a multi-dose reservoir dry powder inhaler containing up to 200 doses. Within the product development work as well as for release control at the manufacturing site, the entire dose interval needs to be accessed. Automated systems are therefore required in the accomplishment of high-quality work at a high-throughput rate. At Astra Draco, the automation of dose measurements started in 1985 and the development of automated methods has since been an important part of our R&D work. The number of robot stations has increased from four stations in 1992 to over 40 identical stations today, located at different sites in Sweden, Germany and USA.

The robot stations are complex instruments which perform a number of tasks including sample handling, dose quantification and evaluation of the results. Different configurations may be used with fast transformation procedures resulting in a highly versatile system. Experiences from the development of the automated systems will be discussed as well as different aspects of quality assurance.

### Automated moisture testing by Karl Fischer titration and other methods

*Colleen S. Nichol, Searle, Stokie, IL, USA*

Moisture testing is a major, high-volume analysis in the pharmaceutical laboratory. Moisture analysis is used in support of stability and formulation development. Moisture content is a very important parameter in the drug development process, because water in the drug product or drug substance can lead to instability, as demonstrated by product degradation, increases or decreases in hardness, friability, etc. Therefore, the product should contain minimal water and be stored in containers so as to reduce or prevent water absorption into the product. The water content is generally tested using a Karl Fischer titration. At Searle, we have successfully automated a Karl Fischer titration workstation. We have investigated several methods that will increase throughput, while at the same time decrease analysts' preparation time. Several different approaches to automating moisture testing of pharmaceutically relevant samples will be discussed. Some of these approaches are manual Karl Fischer titration in a humidity-controlled glove box, the automated moisture system from Bohdan, near-infrared spectroscopic methods, and the Brinkmann moisture system. Data will be presented to show the successes and failures of the various moisture analysis systems examined at Searle.

### A simple customized automated procedure for sample dilution

*Sharon Chang, Ian Harry and Maria Guazzaroni, Pfizer, Control Laboratories, Quality Operations, Brooklyn, NY, USA*

A high-performance liquid chromatography (HPLC) system with electrochemical detections (EC) was utilized to assay a low UV-absorbed drug substance and its dosage forms. Two manual dilutions have to be made for each sample. A Zymark BenchMate II Workstation was evaluated to assist the Quality Operation Laboratory in performing high volumes of sample preparation.

Several factors were considered in developing the procedures for automation.

(i) The HPLC analysis method has a long run time.
(ii) The compound is susceptible to air.
(iii) The dissolving solvent and the diluent contain different organic compositions.
(iv) The final sample volume changes slightly after mixing.
(v) There is an internal standard in the diluent.
(vi) The sample solution contains sugar and has a different density from the standard solution.

A simple dilution procedure using the BenchMate with a volumetric mode, without connecting it to the HPLC system, was designed and validated to replace the manual preparation. Instrument calibration was scheduled to ensure accuracy and precision of the performance. The automated dilution procedure is not only reliable and convenient, but also cost effective and environmentally friendly.

### The evolution of automation and research in regulatory science

*Patrick J. Faustino, Ajaz S. Hussain and Karl P. Flora, Food and Drug Administration Center for Drug Evaluation and Research Division of Product Quality Research, Laurel, MD, USA*

Automation has evolved rapidly since its introduction into the laboratory. Automation or laboratory robotics were used primarily in quality assurance and quality control laboratories, and gradually entered pharmaceutical research laboratories where they currently play an important role in the drug development and drug evaluation functions. In many laboratories, the rate of change is increasing as automation and research priorities are being challenged by advances in technologies that include combinatorial chemistry, novel *in vitro* and *in vivo* assays, and increasingly sophisticated analytical techniques. These challenges may require automation to efficiently evaluate new molecular entities, new preclinical models or novel research endpoints. Changes in research priorities and requirements may require automation to more rapidly evolve to effectively utilize high-throughput techniques to evaluate a complete range of complex issues, e.g. chemistry, pharmaceutics, toxicity, drug metabolism and drug–drug interactions.

The impact of automation and the changes in regulatory research are equally striking. The regulatory research laboratory has evolved from the initial use of laboratory robotics as a tool to address safety issues, e.g. minimizing human exposure to pathological agents, to efficiency issues involving rapid methods development and assay validation as research resources and priorities have changed. Regulatory science has continued its evolution, focusing research efforts to efficiently provide scientific data that can facilitate regulatory decisions. Automation is playing a key role in our laboratory's evaluation of two pre-clinical models, an *in vitro* cell culture model (Caco-2) to determine intestinal permeability of drug substance and drug product, and an *in vivo* transgenic mouse model (TG.AC) to determine the carcinogenicity of drugs. These research efforts supported by intramural automation resources may help in the development of regulatory guidelines that will aid FDA and industry in the NDA process.

### Five-year summary report documenting BenchMate instrument performance preparing 650 urine samples for 2,4-D analysis

*K. K. Brown and J. L. McLaurin, US National Institute for Occupational Safety and Health, Division of Biomedical and Behavioral Science, Cincinnati, OH, USA*

NIOSH conducted an exposure assessment study that required the development and application of a complex clinical chemistry method for the analysis of 2,4-D in urine. One assumption that this methods was developed upon was that robotic automation would free up personal work time in preparing samples, and thus enhance sample throughput and reporting response time. Another assumption was that robotics would improve QC and QC of the analytical data. A BenchMate workstation was used to automate the sample preparation. The robot utilizes an analytical balance accurate to a tenth of a milligram which can be used to track the sample mass before and after each preparation step.

For each 30-sample batch analysed during the study, the mass data output file was transferred from the robot to a spreadsheet and converted into a QC chart. In this QC chart, the volume of liquid used in each sample preparation step was calculated and plotted from the mass record. In this way, sample preparation variables were monitored, including the solid phase extraction cartridge load rinse and elute volumes. Along with charting volume, other parameters were plotted, e.g. reagent transfer efficiency, per cent water added to the organic phase for reversed-phase SPE, and evaporative losses. Thus, every critical parameter of the sample preparation was recorded and monitored. This chart was especially helpful in characterizing the ruggedness of the new method.

By accumulating these batch charts, a BenchMate performance history was charted with each new batch of samples analysed. This performance history documented changes in method parameters, and established a performance baseline on which decisions were made about instrument maintenance and repair. Also, these records provided information for justifying the rejection of 'out of control' data. This information was stored on CD for CLIA documentation.

The BenchMate alone did not improve the response time for delivering exposure data to workers, but it did shift the bottleneck from sample preparation to data reduction. Using the BenchMate did improve QA and QC of sample analysis by documenting the preparation of each sample. Some of the unprecedented advantages of using the BenchMate over manual sample preparation during this study were as follows. (i) Automatic weighing of test tubes allowed for enhanced mathematical analysis of effect variables with respect of their desired response. For example, the effect of SPE loading elution volumes on analyte recovery. (ii) After method development, that same feature allowed the method to be characterized more accurately. It would have been too labour intensive for a chemist to manually weigh and record the mass of a tube before and after every manipulation in the method. (iii) The analytical balance confirmed that the robot can dispense reagents more accurately and precisely than the reported precision for disposable pipettes. (iv) Moreover, the mass record allowed for increased electronic documentation which fulfils the spirit of CLIA'88 that requests high levels of QA and QC for human samples with potential medical diagnostic outcomes.

### The detection and quantitation of drugs in equine serum using Zymark RapidTrace SPE modules

*Robert McKenzie and Gina Dewitt, Michigan Department of Agriculture, East Lansing, MI, USA*

A method was needed to reduce the time involved in the quantitation and confirmation of serum fluorsemide and phenylbutazone and other drugs of interest in equine serum.

Serum samples are placed in test tubes. Urea and buffer spiked with an internal standard are added. The samples are then submitted to solid phase extraction using Zymark Rapid Trace SPE workstations. The samples are eluted directly into appropriately labelled HPLC autosampler vials which are then capped, mixed and analysed, using a C18 column and an HPLC system equipped with diode array and fluorescence detectors.

This method of analysis eliminates most of the tedious and time-consuming steps associated with liquid/liquid extraction and manual SPE procedures, and circumvents the problems associated with the instability of the named drugs when they are being handled at low concentrations. A highly reproducible procedure that requires only the accurate allocation of the initial serum sample will be presented.

### Confirmation of the NIDA five using a single SPE sorbent and an eight-solvent RapidTrace™ system

*Thierry D. Mann and Michael F. Burke, Department of Chemistry, University of Arizona, Tucson, AZ, USA*

The NIDA five is a series of drugs that are most commonly analysed in forensic cases. These drugs include the opiates morphine and codeine, THC, cocaine, PCP, amphetamines, and the barbiturates. It would be ideal if there was one universal method for extracting all drugs. This is not a realistic scenario, however, because many drugs have drastically different properties, and in order for selectivity to prevail, extraction methods must be tailored to certain functionalities of molecules. It is possible, however, to use the single mixed-mode SPE sorbent in combination with eight solvents to achieve high selectivity and efficiency extraction of the NIDA 5.

Although liquid–liquid extraction has been the most common method for sample preparation for forensic analysis, solid phase extraction (SPE) has some distinct advantages. Studies have shown that SPE is more rapid, economical and efficient than LLE. Surveys of NIDA-certified drug-testing labs have shown that approximately 50% of analyses utilize SPE techniques. Cocaine and THC analyses are by far the highest volume assays, and although SPE procedures for these drugs are well accepted, it is not clear that SPE methods for other NIDA 5 are compatible with the solvents used in the previous analyses.

This presentation will address the simplification of SPE procedures using a RapidTrace™ automated system so that no solvent replacement is necessary when changing assays. The use of a single sorbent also simplifies the extraction procedures.

### Steps to automation in a contract research organization

*Robert Massé and Carl Dourambeis, Phoenix International Life Sciences, Montreal, Quebec, Canada*

Phoenix International is one of the fifth largest CROs (contract research organization) in the world, featuring many services for the pharmaceutical industry. Part of Phoenix's portfolio of services, outside clinical research, is a bioanalytical laboratory service consisting of HPLC, GC, GC/MS, HPLC/MS, HPLC/MS/MS and immunochemistry laboratories. Phoenix also has a new drug discovery group consisting of *in vivo*, *in vitro* and analytical drug metabolism areas. Phoenix's service laboratory group typically analyses large volumes of samples requiring routine repetitive manual work.

In today's highly competitive contract research marketplace, there is a strong need for cost reduction and accurate analytical data reporting which reflects on the pricing for the customer. One of the key factors to cost reduction is automation. Although, initially, automation requires an upfront capital expenditure, there will be longer term benefits ariving from this type of investment, including reproducible results, marketing ability of our up-to-date technology, and larger sample throughput with the same staff availability.

Phoenix has established a laboratory group called 'Sample Preparation and Automation (SPA)' to fulfil the necessary tasks of evaluation, implementation and technology transfer of suitable automation workstation platforms and robotic systems. Development of a new type of trust in this technology appears to be one of the larger hurdles to overcome in terms of employee acceptance. Another hurdle comes from the large pharmaceutical companies in terms of method adaptation. Phoenix's challenge is to adapt their developed automated methodology into its internal laboratories. Phoenix needs to meet the clients' requirements for automation technology and still maintain a profitable edge with its capital spending.

Phoenix's initiative in SPA as both a multi-instrument automation development group and a virtual technology transfer group are enabling Phoenix to maintain an edge over the competition by answering the clients' demands for faster, accurate data generation and reporting. This talk will represent the current approach to automation via SPA as well as interdepartmental cooperation, and the challenges encountered. Also presented will be key ideas learned along the way to implement automation technology as well as how we are winning over staff support towards the necessary evolution of automation.

### A comparison of two methods for measuring kinase activity in a high-throughput format

*Maureen Laney, David Lesikar and David Liu, SCIOS, Dept. of Research, Sunnyvale, CA, USA*

Two methods will be compared for measuring kinase activity *in vitro* in a high-throughput format. A primary consideration in choosing a method was for an assay that would be widely applicable to a variety of kinase targets, with minimal changes required when the specific enzyme target changed. One method is an indirect assay which links kinase activity through two auxiliary enzymes to a colorimetric readout. The other method is a radioactive assay with a biotinylated substrate. The product is captured on the SAM$^{2TM}$ streptavidin membrane (Promega). Reactions can be applied to the filter in a high-density array by the use of the Biomek high-density replicating tool (HDRT). Both methods are easily automated for high throughput.

### Re-engineering an automated antiviral drug screening: a novel approach of integrating a high-speed pipetting robot

*Rudy Willebrords and Koen Andries, Janssen Research Foundation, Beerse, Belgium, Eugy Van Gool, Labotec NV, Teralfene, Belgium*

In a continuous effort to optimize our drug screening, we set out new goals to improve reproducibility and increase throughput. After 10 years of faithful service, our robot system had to be replaced and re-engineered.

A central XP-robot installed on a custom-designed track, in a conditioned room (temperature and humidity), remains the heart of the system. A multi-channel high-speed robotic pipettor (Zypettor) was integrated with the central robot to increase the sample throughput. Insufficient number of microplate and tip box positions on the original Zypettor, with its relative slowness, forced us to rebuild this station. We designed a new six-position turntable for microplates and tip boxes to interact with the Zypettor in order to increase the number of microplates as well as the speed of pipetting of the Zypettor. Plates and disposables can be positioned in a wide or narrow orientation to allow rapid pipettng in rows of eight or 12 by the Zypettor. This new dimension and approach may create new opportunities in respect of future configuration developments.

Efforts were also undertaken to expand the capabilities of automated microplate measurements. Spectrophotometric as well as fluorometric assessments were developed to measure the viability of virus- and mock-infected cells. The integration of a multi-reagent addition module (Ras/Ram) and a Biotek 96-well washer were crucial for the realization of those procedures. These experimental implementations result in a flexible robot system designed to run cell-based and enzymatic assays.

### Evaluation of a transplanted Zymate microplate system for 384-well automation

*T. C. Ramaraj, Robotics & Automation—High-Throughput Screening, Wyeth-Ayerst Research, Pearl River, NY, USA*

We will summarize our efforts to implement automated 384-well format assays using a Zymate microplate system. This Zymate microplate system was originally designed and delivered to one of our sister companies for automation of an ELISA format assay in 96-well format. The system was also used for generation of diluted assay plates from predilution plates. The original configuration included a high-speed 12-channel Zypettor module for dilution. In addition, the Zypettor was used for adding substrate to assays and for serial dilution of compounds. The system, with addition of pipetting stations, reagent addition dispensers and an online reader for fluorescent/ spectrophotometric assays. An interesting aspect of this presentation will be evaluation of the Zypettor module for sample addition and small-volume delivery of reagents to 384-well plates. Our attempt to automate a fluorescent assay in the fully integrated system will be supplemented with data collected on throughput, efficiency of plate handling and overall sensitivity of the assay as tailored to the Zymate system.

### Automated cell assays using image analysis

*Thomas Hartmann, Ribozyme Pharmaceuticals, Boulder, CO, USA*

We have developed a number of automated quantitative procedures which allow us to perform cell-based assays, e.g. apoptosis, cytotoxicity, drug delivery, gene expression and proliferation assays. These assays can be used in high-density plates and are amenable to plate readers and robotics. Signal detection using an epifluorescence microscope provides single-cell sensitivity and a dynamic range of at least three orders of magnitude. Automation of the image acquisition and image analysis procedure allows unsupervised scanning of high-density plates. Applications, previously performed with conventional plate readers or fluorescence-activated cell sorters, can take advantage of the instrument's specifications. In particular, counting procedures and colocalization studies should benefit from the increased sensitivity, spatial resolution and multi-parameter capabilities of this instrument.

### Automated analytical characterization of combinatorial libraries by LC/MS

*Olga Issakova, Victor Nikolaev, Shelly Wade, Stefan Walle and Nikolai Sepetov, Selectide, a subsidiary of Hoechst Marion Rousel, Tucson, Arizona, USA*

Combinatorial chemistry was developed several years ago as a tool used by a few companies for rapid identification of new drug candidates. Today it is one of the fastest growing and fastest changing research fields. One of the biggest changes that has occurred in the field within the last few years is the expansion of automation in both synthesis and screening technologies. This change shifted the bottleneck from production of new compounds to their analytical characterization.

Here we present a strategy developed in our lab for automated analysis of libraries synthesized in different formats (low complexity mixtures, arrays of individual compounds, etc.). The goal of analysis is to provide biologists with information about whether or not the desired diversity was achieved, and give information to chemists about the coupling success of the building blocks in each randomization. Approximately 10–20% of the compounds (wells) in a library designed for lead generation, and 100% of compounds synthesized in arrays at the stage of library development or for SAR, are analysed by LC/MS in high-throughput fashion. Examples of the formats of data output used for the analysis will be presented. Aspects of quantitative analysis of combinatorial libraries will be discussed and illustrated utilizing the most recent examples.

### The impact of structure profile libraries on the drug discovery–development continuum

*Robyn A. Rourick and Jeffrey L. Whitney, Bristol-Myers, Squibb Pharmaceutical Research Institute, Wallingford, CT, USA; Kevin J. Volk, Saul W. Fink and Edward H. Kerns, Bristol-Myers Squibb Pharmaceutical Research Institute, New Brunswick, NJ, USA; Mike S. Lee, Bristol-Myers Squibb Pharmaceutical Research Institute, Princeton, NJ, USA*

Considerable emphasis within the pharmaceutical industry has been directed toward acceleration of the drug discovery–pharmaceutical development continuum. In support of these initiatives, greater amounts of information relevant to lead compound selection criteria will serve to accelerate these stages. Combinatorial chemistry is a widely applied approach for the generation of structural diversity and increased numbers of novel compounds with the goal of increasing the rate of novel lead compound discovery. Typically, mixtures of compounds, or 'libraries', having predictable structural variation are tested for activity in a parallel format, thus increasing the probability of discovering active lead compounds. However, reliance on activity alone may not be sufficient for selection of the best pharmaceutical product. Pharmaceutical development issues focused on excipient interactions, chemical stability, metabolic stability, pharmacokinetics, toxicology and chemical process research heavily affect the commercial success of a drug.

The combinatorial approach may be extended for prospective parallel analytical research in pharmaceutical development. Combinatorial predictive development experiments may be conducted in order to rapidly obtain information relevant to early go/no go decisions based on a wide matrix of development information. We have utilized a predictive degradation strategy, aimed at the identification of degradants in advance of their appearance during the drug development process. We employ a strategy using standard LC/MS profiling and LC/MS/MS substructural analysis conditions to rapidly propose the structures of the degradants. Based on these studies, a degradant library is developed which includes information on molecular structures, chromatographic behaviour, molecular weight, UV spectrum and substructure-specific MS/MS product ions of the individual components. This library can then be used as a springboard for the structure dereplication and elucidation of degradation products, impurities and process-related byproducts encountered in samples throughput the drug discovery–development–manufacturing continuum, as well as candidate selection. As a result, in an effort to increase the capacity and throughput of the predictive degradation profiling process, sample preparation was critically evaluated. It was found that sample preparation is often very resource and time consuming, and ultimately a rate-limiting factor. Therefore, as an extension of predictive models, we have developed a procedure for automated sample preparation which involves a Zymate robot. The utilization of these strategies as a means of fulfilling and comparing selection criteria for a number of potential drug candidates will be demonstrated.

### Automated predictive profiling of drug candidates using LC/MS and robotics

*Jeffrey L. Whitney, Robyn A. Rourick and Juan Cadavid, Bristol-Myers Squibb Pharmaceutical Research Institute, Wallingford, CT, USA; Saul W. Fink and Kevin J. Volk, Bristol-Myers Squibb Pharmaceutical Research Institute, New*

*Brunswick, NJ, USA; Mark E. Hail and Mike S. Lee, Bristol-Myers Squibb Pharmaceutical Research Institute, Princeton, NJ, USA*

Recent analytical initiatives aimed at accelerating the drug discovery and early development continuum have focused on obtaining greater amounts of structural information on potential drug candidates earlier in the process. In our pharmaceutical research and development laboratories, analytical strategies based on standard and automated LC/MS methods [Rourick, R. A. *et al.*, *10th AAPS Annual Meeting Proceedings* (1994), Kerns, E. K. *et al.*, *J. Nat. Prod.*, **57**, 1391 (1994)] are highly utilized to rapidly provide this information.

Critical factors affecting the early development of a drug include chemical stability, metabolic stability, formulation development, process optimization and safety. Traditionally, these factors are only addressed in a 'just in time' capacity during development, quite often negatively affecting critical timelines. One proactive use of our LC/MS methods involves performing predictive development profiles [Thompson, W. T. *et al.*, *Proceedings of the 43rd ASMS Conference*, 174 (1995)] on selected drug candidates during the discovery process. This approach involves intentional chemical transformation of a potential drug with a variety of sample conditions followed by LC/MS and LC/MS/MS analysis to elucidate any new components. Structures resulting from the transformation of a potential drug are assembled into a structure profile library (SPL) along with relevant source and reaction parameters. Subsequently, the SPL is made available to all downstream development areas for reference throughout the lifetime of the drug candidate. In this way, components in samples generated throughout preclinical development and Phase I–IV studies can be rapidly identified by referencing the SPL.

Current initiatives aimed at accelerating the predictive profiling process involve automated analysis methods, information management systems, combinatorial incubation experiments [Volk, K. J. *et al.*, *Proceedings of the 44th ASMS Conference*, 176 (1996)] and integrated robotic methods [Whitney, J. L. *et al.*, *15th ISLAR Conference Proceedings* (1997)]. These initiatives address the current rate-limiting steps, which include sample production, sample introduction, structure analysis and data management. Examples will be shown illustrating our LC/MS-based predictive profiling methods and integrated robotic method development station.

### Automation and computer design in chemical synthesis: a small company perspective

*Paul M. Doyle, Stephen R. Hopkins and Emma Barker, BioFocus, Sittingbourne Research Centre, Sittingbourne, Kent, UK*

Currently, there are a bewildering array of technological solutions to the general problem of enhancing the throughput of chemical synthesis in the pharmaceutical and related industries. The degree of sophistication in chemical manipulation that can be achieved is impressive, although this complexity carries the penalty of cost. For the smaller laboratory, not only must there be a wide variety of techniques available to the chemist, but they must be versatile and represent good value for the investment made. At BioFocus, we are essentially a service-based, problem-solving organization, which means that we are presented with a wide range of other people's starting points. In order to meet tight deadlines, we cannot afford to be locked to one technology, but must quickly make an assessment of the correct technique for the problem to hand. We use computation experimental design to constrain the number of chemicals we need to synthesize in order to achieve maximal information (predictive array design™). We have a variety of automated and semi-automated synthetic methodologies to provide both solid phase and solution syntheses, and have constrained ourselves to high-quality end products.

Understanding the physical and chemical limitations of the synthetic and analytical techniques allows integration when appropriate. These issues will be addressed in the talk, illustrated with practical examples.

### Systems development strategy: a component-based approach, the overview

*Dinesh S. Thakur, Vincent W. Chen, Robi L. Banerjee, Matthew G. Holt and Kirk J. Leister, Pharmaceutical Research Institute, Bristol-Myers Squibb Company, Syracuse, NY, USA*

A component-based framework has been developed which facilitates software re-use and provides faster integration of laboratory automation systems. Implementation of laboratory automation systems by the traditional approach usually takes significant development time as both module-level and application-level software are written custom to a particular application. In addition, modification and adaptation of an existing system developed by the traditional approach, to new and enhanced applications, usually requires significant reconfiguration to system software. This is because modification and reconfiguration affect the way in which the module-level software interacts with each other. In order to reduce the development time, and build flexible and easily re-configurable systems, a component-based framework has been developed that addresses the issue of software re-use and run-time configuration. This framework is based on a services-based architecture and supports stateless components that can be configured at run-time. In addition, this framework supports polymorphic behaviour for all its components, which allows for integrated substitution of hardware offering similar functions into an existing system to enhance its functionality. Run-time configuration has an additional benefit of making laboratory automation systems fault tolerant. This presentation outlines the basic overview of this framework in the context of laboratory automation systems, introduces the concepts involved and provides a high-level description of the architecture used to develop this framework.

### Systems development strategy: a component-based approach, the architecture

*Vincent W. Chen, Matthew G. Holt, Robi L. Banerjee, Dinesh S. Thakur and Kirk J. Leister, Pharmaceutical Research Institute, Bristol-Myers Squibb Company, Syracuse, NY, USA*

The technical detail of the architecture for laboratory automation systems built using the component-based framework will be described in this presentation. The interactions between the system coordinator, the system repository, system scheduler, system logger and other peripheral components will be presented in the context of the underlying services-based architecture. Description of the communication interfaces between various components of an automated system will be explained. Several design patterns were used to develop this architecture. The influence of these patterns on the design of laboratory automation systems will be addressed. Also, the influence of unified modelling language on interface design will be discussed. Integration of the system components with the peripheral components to build an automated system based on this framework will be presented with emphasis on run-time configuration and component reuse. In addition, the integration of this architecture with enterprise applications, e.g. MS Exchange and MS IIS will be addressed. The development of a laboratory component based on this framework will also be described.

### Systems development strategy: a component-based approach, the prototype

*Robi L. Banerjee, Matthew G. Holt, W. Chen, Dinesh S. Thakur and Kirk J. Leister, Pharmaceutical Research Institute, Bristol-Myers Squibb Company, Syracuse, NY, USA*

This presentation provides a concrete example of an application developed using the component-based framework. A sample inventory and control system was developed for the purpose of aliquoting, distributing and tracking biologic molecules in pharmaceutical drug development. This presentation will provide an overview of the workflow involved, a detailed description of the functional implementation of the robotic aliquoting system, and a demonstration of the application with the real world implementation of the component-based framework.

### Accelerated discovery of cysteine protease inhibitors by the high-throughput bioassay of α-ketoamide-based compounds created by automated parallel synthesis

*H. Cheng, M. Cullinane, K. Brinner, C. Baldino and S. Chipman, Departments of Biology and Process Chemistry, ArQule, Medford, MA, USA; M. Laboissiere and C. Craik, Department of Pharmaceutical Chemistry, University of California at San Francisco, San Francisco, CA, USA*

Cysteine proteases have been implicated in a wide variety of human pathophysiological conditions. This class of enzymes can be subdivided into $> 14$ different families on the basis of sequence homology, the calpain and papain families being the most significant. We chose a papain-like cysteine protease, cruzain, to demonstrate how the integration of automated, parallel chemical synthesis and high-throughput screening has accelerated the discovery and optimization of small organic compound inhibitors. Cruzain is a protease isolated from *Trypanosoma cruzi*, the causative agent in Chagas' disease. The peptidyl α-ketoamide class of compounds are known to be both cysteine and serine protease inhibitors. Using solution phase, combinatorial unit synthesis was created $\sim$45 000 discrete α-kitoamide compounds (via the selective monoacylation of diamines) that were arrayed in a spatially addressable, 96-well microtitre plate format. To address the effectiveness of this class of compounds against cysteine proteases, we tested their ability to inhibit cruzain. Initial results yielded a series of three lead compounds with sub-μM $IC_{50}$ activity and up to $>$ 10-fold selectivity versus cathepsin B and a panel of serine proteases. The structure–activity relationships observed from the initial screening of the spatially addressable α-ketoamide array facilitated the design and synthesis of additional compounds, that yielded further increases in potency and specificity. The integration of spatially addressable chemical synthesis and high-throughput screening accelerated the process of inhibitor discovery and optimization for the protease cruzain.

### High-throughput screening—a multidisciplinary team effort

*A. E. Fletcher, M. S. Briggs, G. Foster, J. Kerby and N. Allen, Merck Sharp and Dohme, Neuroscience Research Centre, Terlings Park, Eastwick Road, Harlow, Essex, UK*

The increasing demands on the pharmaceutical industry to enhance the drug discovery process has led to the introduction of automated high-throughput screening as an 'industry standard'. However, the widespread implications of establishing such systems is frequently overlooked by many companies and can be in some cases the limiting factor to efficient HTS. Any successful HTS operation requires a dedicated and committed multidisciplinary team to ensure efficient maximum productivity. At the Neuroscience Research Centre we have established such a team which encompasses representatives from scientific (Sample Repository, HTS and Tissue Culture) and support (Research Engineering, Purchasing and IT) staff. The close interaction between the departments and the benefits of such a team will be reviewed together with an overview of the strategy employed for our HTS program.

### Maximizing assay capability of older robotic systems by incremental introduction of new technology to the screening laboratory

*M. Elizabeth Miller, Rohm and Haas Company, Spring House, PA, USA*

The Agricultural Discovery Department at Rohm and Haas Company has used a Zymark robotic workcircle to perform fungitoxicity screening in microtitre plates for about 10 years. At the time of delivery in 1988, the system was considered state-of-the-art for handling microplate assays, and was comprised completely of custom-designed stations, as microplate-handling devices were almost unknown in those days. Since then, microplate handling on integrated robotic workcircles has become routine, and a variety of 'off-the-shelf' plate handling stations are now available from different vendors, obviating the need for custom stations in many cases. In light of new developments in screening technology, do older integrated robotic systems still have a place in the laboratory? Depending upon the demands on the screening organization, the answer can certainly be yes. We are still using our original workcircle with custom stations, with only some minor upgrades made to the system. In order to accommodate newer assay procedures, however, we have added several stand-alone workstations to the laboratory, which are used for a variety of work processes not manageable on our Zymark system. This combined approach of using an integrated workcircle in concert with stand-alone units has been a very cost-effective way of enhancing our screening capabilities while continuing to make full use of our investment in our original robotic system.

### Information—not just data: use of a robotic dissolution system to provide rapid graphical feedback to formulators

*Mark J. Dryfoos, Stability, Robotics & Automation Group, PHAD/ARD Novartis Pharmaceuticals Corporation, East Hanover, NJ, USA*

Early in the development stage of a solid dosage form, many variants are produced. The sheer number of samples to be tested can be overwhelming as formulators try to achieve an optimal balance of lubricants, disintegrates, coating thickness, tablet hardness, etc. Because these samples will neither be used in clinical trials nor in primary stability studies, a less formalized approach (i.e. non-GMP) to their dissolution testing may be utilized.

We have adopted just such a 'second-track' strategy in the Stability, Robotics, & Automation Group, both to ease the burden on the GMP Dissolution Testing Lab, as well as to provide rapid turnaround, high value-added results to the formulators. This capability is provided by an automated Apparatus 2 dissolution system featuring a Zymate XP robot with online fast HPLC. Customized spreadsheets, macros and presentation graphics templates allow for rapid data reduction and plotting. Several unique aspects of the system as well as the advantages of this approach will be discussed and illustrated.

### An automated assay for ligands of nuclear receptors

*Richard K. Harrison, Litao Zhang, Ken Locke and Geoff Schwartz, Rhone-Poulenc Rorer, Collegeville, PA, USA*

Nuclear receptors are ligand-activated transcription factors. Their function in regulation of gene expression makes them attractive targets for therapeutic intervention in various metabolic diseases. As part of an ongoing program aimed at regulation of these receptors, we have developed a high-throughput assay for determination of compounds than can bind to nuclear receptor targets of interest. The assay is a simple homogeneous system based on scintillation proximity protocols, different from other SPA assays for nuclear receptors in the use of underivitized ligand binding domain, and is capable of screening over 50 000 compounds/week. The evolution, from a low-throughput manual assay, to semi-automated, and finally to the development of the UHTS used for several nuclear receptors will be discussed.

### Generic testing procedure for automated analytical chemistry workstations

*Craig Bender, Searle R&D, Skokie, IL, USA*

### Automation of liquid handling for high-throughput screening in 1536-well microplates with the Robbins Hydra and automated plate positioning system

*David W. Batey and Jim Stanchfield, Robbins Scientific, Sunnyvale, CA, USA*

Distribution of compounds and dispensing of reagents used in high-throughput screening assays are readily performed with Hydra microdispensers. The 96 (or 384) glass syringes of the Hydra are held in a fixed array positioned at the centre of each well in a standard microplate. Precision mechanical components allow an accuracy of +1% for dispensing 1 μl with a coefficient of variation of less than or equal to 3% across the syringe array. With syringes available in sizes ranging from 100 μl to 1 ml, the Hydra is well suited for transfer of bulk reagents as well as distribution of compounds in submicrolitre volumes down to 100 nl. When equipped with the newly developed automated plate positioner, the Hydra-96 and Hydra-384 are ideally suited for high-throughput, repetitive dispensing procedures. All plate and liquid handling functions can be controlled through user-defined parameters within the included software or through simple ASCII commands when used in an integrated system. The stage can accommodate a combination of two plates of various well densities with a third location dedicated as a wash reservoir that is filled and emptied by peristaltic pumps. Settings for the positioning of the plates during dispense/aspirate operations can be adjusted in increments of 5 μm. This level of positional accuracy allows consistent and reproducible access into current 1536-well microplates as well as future high-density formats. For reformatting operations, any combination of 96- (Hydra-96 only), 384- and 1536-well microplates can be placed in the two plate positions and re-arrayed through software protocols defined by the user. Fluorescence signal was obtained for reactions assembled and distributed into all wells of 1536-well plates. By following recommended wash protocols for the glass syringes, the results indicated no carryover contamination between samples or reagents. Reaction components included compounds stored in 100% DMSO as well as aqueous reagents. Process time for reaction assembly in 1536-well plates will be presented. The Hydra-96 and Hydra-384 microdispensers, equipped with the automated plate positioner stage and software make up a complete high-throughput system for accurate, precise dispensing into today's high-density microplates.

